# ADP Ribosylation Factor 4 (Arf4) Regulates Radial Migration through N-Cadherin Trafficking during Cerebral Cortical Development

**DOI:** 10.1523/ENEURO.0125-23.2023

**Published:** 2023-11-07

**Authors:** Yoshinobu Hara, Takehiko Katsuyama, Masahiro Fukaya, Takeyuki Sugawara, Tomoko Shiroshima, Tetsushi Sadakata, Noriko Osumi, Hiroyuki Sakagami

**Affiliations:** 1 Department of Anatomy, Kitasato University School of Medicine, Sagamihara, Kanagawa 252-0374, Japan; 2 Education and Research Support Center, Gunma University Graduate School of Medicine, Maebashi, Gunma 371-8511, Japan; 3 Department of Developmental Neuroscience, Tohoku University Graduate School of Medicine, Sendai, Miyagi 980-8575, Japan

**Keywords:** Arf, membrane traffic, N-cadherin, radial migration, small GTPase

## Abstract

During the development of the cerebral cortex, N-cadherin plays a crucial role in facilitating radial migration by enabling cell-to-cell adhesion between migrating neurons and radial glial fibers or Cajar–Reztius cells. ADP ribosylation factor 4 (Arf4) and Arf5, which belong to the Class II Arf small GTPase subfamily, control membrane trafficking in the endocytic and secretory pathways. However, their specific contribution to cerebral cortex development remains unclear. In this study, we sought to investigate the functional involvement of Class II Arfs in radial migration during the layer formation of the cerebral cortex using mouse embryos and pups. Our findings indicate that knock-down of Arf4, but not Arf5, resulted in the stalling of transfected neurons with disorientation of the Golgi in the upper intermediate zone (IZ) and reduction in the migration speed in both the IZ and cortical plate (CP). Migrating neurons with Arf4 knock-down exhibited cytoplasmic accumulation of N-cadherin, along with disturbed organelle morphology and distribution. Furthermore, supplementation of exogenous N-cadherin partially rescued the migration defect caused by Arf4 knock-down. In conclusion, our results suggest that Arf4 plays a crucial role in regulating radial migration via N-cadherin trafficking during cerebral cortical development.

## Significance Statement

In the cortical layer formation, the distribution of N-cadherin on cell surface in migrating neurons is tightly regulated by endosomal trafficking system. However, its molecular detail remained fully understood. Here, we demonstrated that ADP ribosylation factor 4 (Arf4) small GTPase, a critical regulator of membrane trafficking in the *trans*-Golgi network (TGN), plays distinct roles from Arf5 in radial migration. We further demonstrated that Arf4 regulates N-cadherin trafficking in and out of the TGN and the contact of migrating neurons with radial fibers. Our results suggest that Arf4 regulates radial migration through N-cadherin trafficking in the TGN.

## Introduction

The six-layered neocortex is a unique feature of mammals, responsible for higher brain functions such as cognition, sensory perception, emotion, learning, and memory. Glutamatergic projection neurons are generated from progenitor cells in the ventricular zone (VZ) and subventricular zone (SVZ) of the dorsal pallidum and undergo radial migration to their final cortical layers in an inside-out pattern (Lui et al., 2011; Buchsbaum and Cappello, 2019; Jossin, 2020). This migration process comprises three distinct migratory modes: multipolar migration in the intermediate zone (IZ; Tabata and Nakajima, 2003), radial glia-guided locomotion in the cortical plate (CP; Rakic, 1972; Nadarajah et al., 2001), and terminal translocation through the primitive cortical zone of the upper CP (Nadarajah et al., 2001; Sekine et al., 2011). Defective radial migration can lead to congenital cortical malformations such as lissencephaly, periventricular nodular heterotopia, and subcortical band heterotopia (Kato and Dobyns, 2003; Friocourt et al., 2011). These conditions are also associated with various neurodevelopmental disorders (Hamada et al., 2015; Packer, 2016; Katayama et al., 2017; Guidi et al., 2018; Ossola and Kalebic, 2021; Sokpor et al., 2022). Therefore, understanding the mechanisms that regulate radial migration is crucial for developing potential treatments for these disorders.

During radial migration, neurons undergo dynamic changes in their cell shapes and migrate in a specific direction by extending processes and sensing environmental cues through cell-cell and cell-extracellular matrix adhesions (Kawauchi, 2015; Peyre et al., 2015; Martinez-Garay, 2020). N-cadherin, a calcium-dependent adhesion molecule of the classical cadherin family, mediates almost every step of radial migration through cell-cell adhesion between migrating neurons and radial glial fibers or Cajar–Reztius cells (Gil-Sanz et al., 2013; Martinez-Garay et al., 2016). Accumulating evidence has revealed that membrane trafficking of N-cadherin plays a crucial role in radial migration by regulating the spatiotemporal expression of surface N-cadherin in migrating neurons (Kawauchi et al., 2010; Wu et al., 2016; Hor and Goh, 2018). However, our understanding of the molecular mechanisms by which membrane trafficking controls N-cadherin-dependent radial migration remains incomplete.

The ADP ribosylation factor (Arf) small GTPases are crucial for regulating membrane trafficking and maintaining organelle integrity. Arfs function as molecular switches that cycle between GDP-bound and GTP-bound states, and their conversion to the GTP-bound state by Sec7 domain-containing guanine nucleotide exchange factors (GEFs) induces conformational changes, allowing them to recruit effector proteins and to activate lipid-modifying enzymes, thereby facilitating various steps of membrane trafficking (D’Souza-Schorey and Chavrier, 2006; Donaldson and Jackson, 2011). The canonical Arf family comprises six members (Arf1–6) in mammals (Kahn et al., 2006), which can be structurally divided into three classes: Class I (Arf1–3), Class II (Arf4–5), and Class III (Arf6). Concerning the roles of Arfs in cortical development, mutations in human genes for ARF1 and its GEF, ARFGEF2, have been linked to periventricular nodular heterotopia, suggesting the involvement of Arf1 in cortical radial migration (Sheen et al., 2004; Gana et al., 2022). Arf6 also regulates multipolar migration, multipolar-to-bipolar transition in the IZ and N-cadherin recycling in migrating neurons in rodents (Falace et al., 2014; Hara et al., 2016). However, the functional roles of Class II Arfs in cortical development are not yet fully understood, as they have been considered supplementary or redundant to Arf1 because of their high sequence similarity and overlapping localization to the Golgi. However, recent evidence suggests that Arf4 has unique functions in cellular processes, such as the transport of rhodopsin in photoreceptors and Notch components in differentiating keratinocytes (Deretic et al., 2005; Ezratty et al., 2016). Genetic deletion of Arf4 in mice results in mid-gestational lethality, likely because of growth retardation by dysfunction of the visceral endoderm (Follit et al., 2014). In terms of neuronal functions, heterozygous deletion of Arf4 in mice results in impaired dentate gyrus-dependent pattern separation with reduced spine density in the dentate gyrus (Jain et al., 2012), whereas Arf4^+/−^/Arf5^−/−^ mice exhibit essential tremor-like behaviors with impaired targeting of Nav1.6 to the axon initial segment in cerebellar Purkinje cells (Hosoi et al., 2019).

To elucidate the role of Class II Arfs in cortical development, we first examined the cellular and subcellular localization of Class II Arfs in the developing cerebral cortex by immunohistological analyses using isoform-specific antibodies. We then examined the effect of Arf4 and Arf5 knock-down on cortical layer formation, cell morphology and Golgi orientation in the migrating neurons in the IZ, and their migration speed in both the IZ and CP. Furthermore, we compared N-cadherin subcellular localization and the morphology of cell organelles between Arf4-knock-down and control migrating neurons. Our results provide the first evidence for specific roles of Arf4 in cortical radial migration through N-cadherin trafficking.

## Materials and Methods

### Experimental animals

All experimental procedures in this study were approved by the Animal Experimental and Ethics Committee of The Kitasato University School of Medicine (#2018–138, #2019–138, and #2020–138). Pregnant ICR (Institute of Cancer Research) mice were purchased from The Jackson Laboratory Japan. Mice were maintained in standardized pathogen-free conditions with 12/12 hour (h) light/dark cycle at room temperature with at libitum access to food and water at the Center for Genetic Studies of Integrated Biological Functions of Kitasato University School of Medicine. Mouse embryos and pups of either sex were used in the experiments.

### Plasmids construction

The cDNAs for Arf4, Arf5, and syntaxin 16 (STX16) were amplified by a PCR from a mouse embryonic day (E)17 brain cDNA library using advantage HF2 polymerase (Takara Bio Inc.) and the following primer sets supplemented with the EcoRI or SalI restriction enzyme recognition sequence (underlined): 5′-ACC ATG GGC CTC ACC ATC TCC TCT CTC-3′ and 5′-GAA TTC ACG TTT TGA AAG TTC ATT TGA CAG CCA AT-3′ for Arf4; 5′-ACC ATG GGC CTC ACG GTG TCC GCG CTC-3′ and 5′-GAA TTC GCG CTT TGA CAG CTC GTG-3′ for Arf5; 5′-GTC GAC AAT GGC CAC CAG GCG TTT AAC CGA CG-3′ and 5′-CTA GCG AGA CTT TAC GGC GAC GAG G- 3′ for STX16. The PCR fragments were cloned into pGEM-T Easy vector (Promega). Point mutations were introduced using the PrimeSTAR Mutagenesis Basal kit (Takara Bio Inc.) with following primer sets; 5′-GTT GAC AGT AAC GAC CGT GAA AGA ATC-3′ and 5′-GTC GTT ACT GTC AAC CAC AAA AAT GAG-3′ for short hairpin RNA (shRNA)-resistant wild-type Arf4. Each Arf cDNA fragment was digested with EcoRI and subcloned in frame to the upstream of C-terminal hemagglutinin (HA) epitope sequence in the pCAGGC vector (Niwa et al., 1991), whereas STX16 cDNA fragment was digested with SalI and NotI and subcloned to the downstream of the FLAG epitope sequence in the pCAGGS vector (Niwa et al., 1991; Hara et al., 2016). To construct shRNA vectors (shArf4, shArf5, and control), oligonucleotides targeting mouse Arf4 (#1, 5′-TGG TAG ATA GCA ATG ATC GTG-3′; #2, 5′-TCT GGA AGA TGA GCT GCA G-3′), Arf5 (5′-TCT GCT GAT GAA CTC CAG A-3′), or firefly luciferase (5′-CGT ACG CGG AAT ACT TCG A-3′) aligned with their complementary sequence in tandem by a hairpin loop sequence (5′-TTC AAG AGA-3′) were inserted into the mU6pro vector (Yu et al., 2002; Kawauchi et al., 2010). All plasmids were purified with the PureLink HiPure Plasmid Filter Purification kit (Thermo Fisher Scientific).

### *In utero* electroporation (IUE)

IUE was performed on embryonic day 14 (E14) as described previously (Tabata and Nakajima, 2001; Kawauchi et al., 2003). Each plasmid was dissolved in PBS with 1% of fast green as follows; 1 μg/μl shRNA vector for knock-down experiments; 1 μg/μl shRNA vector and 0.1 μg/μl pCAGGS-shRNA-resistant Arf4 for rescue experiments, together with 0.5 μg/μl pCAGGS-enhanced green fluorescein protein (EGFP) or pCAGGS-mCherry. Timed pregnant mice were deeply anesthetized with inhalation of 2–3% isoflurane, and uterine horns were exposed. After 1–2 μl of plasmid solution was injected into the lateral ventricle through the uterus wall by a glass needle, embryos at E14 were subjected to electroporation (35 V, 450 ms, four pules) using a square electroporator (CUY21EDIT, BEX. Co, Ltd.) and forceps type of electrodes (LF650P3-5, BEX. Co, Ltd.). Embryos or neonates were fixed with 4% paraformaldehyde by transcardial perfusion at 1 d (E15), 3 d (E17), or 5 d [postnatal day 0 (P0)] after electroporation. To label mitotic cells, 10 mg/ml of 5-bromo-2’-deoxyuridine (BrdU; Roche) dissolved in PBS was injected intraperitoneally to E15 pregnant mice at 140 mg/kg body weight three times in 5-min intervals before being killed (Stoykova et al., 1997; Hara et al., 2013).

### Cell culture and transfection

To evaluate the efficiency of shRNAs, primary cortical neurons were prepared from E14 mouse embryos, as described previously (Hara et al., 2016). Before plating, cortical neurons were transfected with shRNA plasmids and pCAGGS-EGFP by electroporation (Amaxa Nucleofector 2D, Lonza) according to the manufacturer’s protocol. Three days after plating, neurons were subjected to immunoblotting with antibodies against Arf4, Arf5, and α-tubulin.

### Antibodies

Antibodies used in this study were summarized in Table 1. An anti-STX16 antibody was raised by immunizing a rabbit with a keyhole limpet hemocyanin (KLH)-conjugated 15-aa peptide (CSLDPEAAIGVTKRS), which corresponded to amino acids 61–74 of rat STX16. For characterization of the anti-STX16 antibody, the total lysate of adult mouse brains was prepared as previously (Sakagami et al., 2013). HEK293T cells were transfected with pCAGGS-FLAG-STX16 using polyethylenimine Max (Polyscicences) and harvested with 2× SDS sample buffer (125 mm Tris-HCl [pH 6.8], 4% SDS, 20% glycerol, 1% sodium deoxycholate, and 10% 2-mercaptoethanol) at 1 d after transfection. After boiling for 5 min, the lysates of brains (10 μg) and HEK293T cells were to immunoblotting with the anti-STX16 antibody and anti-FLAG IgG.

**Table 1:** List of antibodies used in this study

Antibody	Concentration		Species	Antigen retrieval	Source	Catalog number	RRID
	(IHC)	(WB)					
Primary							
Anti-Arf4	1:200	1:1000	Rabbit polyclonal		Hosoi et al. (2019)		
Anti-Arf5	1:200	1:1000	Rabbit polyclonal		Hosoi et al. (2019)		
Anti-α-Tubulin		1:3000	Mouse monoclonal		Sigma-Aldrich	MABT205	AB_11204167
Anti-BLBP	1 μg/ml		Goat polyclonal		Nittobo Medical Co Ltd.	MSFR100290	AB_2571664
Anti-BrdU	1 μg/ml		Mouse monoclonal	2 N HCl, 37°C, 10 min	BD Transduction Laboratories	3D4	AB_2033929
Anti-Cux1	1 μg/ml		Rabbit polyclonal	CA (pH 6.0), 75°C, 1 h	Santa Cruz Biotechnology	sc-13024	AB_2261231
Anti-EEA1	1 μg/ml		Rabbit polyclonal		Fukaya et al. (2014)		AB_3065105
Anti-EGFP	1 μg/ml		Rabbit polyclonal		Sakagami et al. (2005)		
Anti-EGFP	1 μg/ml		Guinea pig polyclonal		Sakagami et al. (2005)		
Anti-EGFP	1 μg/ml		Chicken polyclonal		Aves labs	GFP-1020	AB_10000240
Anti-FLAG(M2)		1:3000	Mouse monoclonal		Sigma-Aldrich	F3165	AB_259529
Anti-GM130	1 μg/ml	1:1000	Mouse monoclonal		BD Transduction Laboratories	610822	AB_398141
Anti-HA	1 μg/ml		Rat monoclonal		Sigma-Aldrich	3F10	AB_2314622
Anti-mCherry	1 μg/ml		Rabbit polyclonal		Hara et al. (2013)		
Anti-mCherry	1 μg/ml		Guinea pig polyclonal		Hara et al. (2013)		AB_2827679
Anti-mCherry	1 μg/ml		Chicken polyclonal		Applied Biologicalalal Materials	Y030151	
Anti-N-cadherin	1 μg/ml		Guinea pig polyclonal		Hara et al. (2016)		
Anti-N-cadherin	1 μg/ml		Rabbit polyclonal		Takara Bio company	M142	
Anti-NeuN	1:1000		Mouse monoclonal	CA (pH 6.0), 75°C, 1 h	Chemicon	MAB377	AB_2298772
Anti-PSA-NCAM	1:500		Mouse monoclonal		Seki and Arai (1991)		AB_2315215
Anti-Rab11	1:250	1:1000	Mouse monoclonal	TE (pH 9.0), 75°C, 1 h,	BD Transduction Laboratories	610657	AB_397984
Anti-Sox2	1 μg/ml		Rabbit polyclonal	CA (pH 6.0), 75°C, 1 h	Proteintech		
Anti-STX12	1 μg/ml		Rabbit polyclonal		Hara et al. (2013)		
Anti-STX12	1 μg/ml	1:1000	Guinea pig polyclonal		Hara et al. (2013)		
Anti-STX16	1 μg/ml	0.1 μg/ml	Rabbit polyclonal		This study		
Anti-TGN38A	1 μg/ml	1:1000	Guinea pig polyclonal		Ibuchi et al. (2020)		
Anti-Tuj1	1 μg/ml		Guinea pig polyclonal		Nittobo Medical Co Ltd.	MSFR105990	
Anti-VAMP4	1 μg/ml	1:1000	Mouse monoclonal	TE (pH 9.0), 75°C, 1 h,	Proteintech	67219-1-Ig	AB_2882510
Secondary							
Anti-guinea pig IgG Alexa 488	1:300		Donkey polyclonal		Jackson ImmunoResarch	706-545-148	AB_2340472
Anti-guinea pig IgG Alexa 594	1:300		Donkey polyclonal		Jackson ImmunoResarch	706-585-148	AB_2340474
Anti-guinea pig IgG Alexa 647	1:300		Donkey polyclonal		Jackson ImmunoResarch	706-605-148	AB_2340476
Anti-guinea pig IgG Alexa 594	1:300		Donkey polyclonal		Jackson ImmunoResarch	703-545-155	AB_2340375
Anti-guinea pig IgG Alexa 647	1:300		Donkey polyclonal		Jackson ImmunoResarch	703-585-155	AB_2340377
Anti-guinea pig IgG HRP	1:10,000		Donkey polyclonal		Jackson ImmunoResarch	706-036-148	AB_2340448
Anti-goat IgG Alexa 594	1:300		Donkey polyclonal		Jackson ImmunoResarch	705-585-147	AB_2340433
Anti-mouse IgG Alexa 488	1:300		Donkey polyclonal		Thermo Fisher Scientific	A-21202	AB_141607
Anti-mouse IgG Alexa 594	1:300		Donkey polyclonal		Thermo Fisher Scientific	A-21203	AB_141633
Anti-mouse IgG Alexa 647	1:300		Donkey polyclonal		Jackson ImmunoResarch	715-605-151	AB_2340863
Anti-mouse IgG HRP	1:10,000		Sheep		GE Healthcare	NA931	AB_772210
Anti-rabbit IgG Alexa 488	1:300		Donkey polyclonal		Thermo Fisher Scientific	A-21206	AB_2535792
Anti-rabbit IgG Alexa 594	1:300		Donkey polyclonal		Thermo Fisher Scientific	A-21207	AB_141637
Anti-rabbit IgG Alexa 647	1:300		Donkey polyclonal		Thermo Fisher Scientific	A-31573	AB_2536183
Anti-rabbit IgG HRP	1:10,000		Donkey polyclonal		GE Healthcare	NA934	AB_772206

CA, citrate buffer; HRP, horseradish peroxidase; TE, Tris-EDTA.

### Immunoblotting

Cortical neurons were harvested with a buffer consisting of 50 mm Tris-HCl (pH 7.5), 150 mm NaCl, 30 mm MgCl_2_, 1% Triton X-100, 10% glycerol, and a cocktail of protease inhibitor (Roche), and then dissolved with 2× SDS sample buffer. After boiling at 95°C for 5 min, 10 μg of lysates were electrophoretically separated on SDS-polyacrylamide gels and transferred onto polyvinylidene difluoride (PVDF) membranes. The blots were incubated with antibodies against Arf4, Arf5, or α-tubulin. After incubation with horseradish peroxidase-linked species-specific secondary antibody (Table 1), immunoreactive bands were detected using the ECL-Plus Western Blotting Detection kit (Thermo Fisher Scientific) and an image analyzer (GE HealthcareImager 680, Cytiba). The optical density of each immunoreactive band was quantified from three independent blots using Fiji, an open-source image processing software (Schindelin et al., 2012; RRID: SCR_002285).

### Immunohistology

Fixed brains immersed in 30% sucrose were sectioned at a thickness of 20 μm using a cryostat (CM3050S, Leica Biosystems GmbH) and hydrated with PBS containing 0.1% Tween 20 (PBST) for three times. Then, the sections were incubated with blocking buffer containing 3% bovine serum albumin (Sigma-Aldrich) and 0.5% Triton X-100 for 1 h and incubated with primary antibodies listed in Table 1 overnight. The sections were washed with PBST for three times and incubated for 3 h with species-specific secondary antibodies (Table 1). Nuclei were counterstained with 4’6,-diamidino-2-phenylindole (DAPI; Roche). Coverslip was mounted using Fluoromount (Diagnostic BioSystems Inc.). Antigens were retrieved by incubation with 0.01 m citrate buffer [0.01 m trisodium citrate dihydrate (pH 6.0) and 0.5% Tween 20] for 1 h at 75°C, or Tris-EDTA (TE) buffer (pH 9.0) for 1 h at 75°C (Table 1). Detection of BrdU was performed as described previously (Hara et al., 2010). Immunoreactions were investigated using a confocal laser microscopy (LSM 710, LSM980 Airyscan, Carl Zeiss).

### Real-time imaging

Real-time imaging was performed as described previously (Tabata and Nakajima, 2003). To label cells sparsely, we used conditional expression plasmids and a low concentration of the Cre recombinase expression plasmid. Embryos were electroporated with shRNA plasmid plus pCAGGS-FloxP-EGFP (1 μg/μl; Shitamukai et al., 2011), pCAGGS-FloxP-farnesylated EGFP (EGFP-F; 1 μg/μl; Shitamukai et al., 2011), pCAGGS-FloxP-mCherry-NLS (1 μg/μl; Hara et al., 2016), and pCAGGS-Cre (0.1 μg/μl; Shitamukai et al., 2011) at E14, killed at E17, and subjected to organotypic brain slice. Recording was performed using a confocal laser microscopy (LSM710, Carl Zeiss) and stage top incubator (40% O_2_, 5% CO_2_; ZILCS-H3, TOKAI HIT), and images were captured every 15 min for 20 h. Migration speed was analyzed using Fiji software. Multipolar migrating cells in the IZ was distinguished from locomoting neurons by their position, cell morphology, and migration behaviors, such as direction and speed, as described previously (Tabata and Nakajima, 2003).

### Quantitative analysis

Colocalization coefficient of Arf4, Arf5, or N-cadherin with several organelle markers was analyzed using ZEN software (Carl Zeiss; RRID: SCR_013672). The contours of EGFP-labeled transfected migrating neurons in the upper IZ at E17 were outlined by segment line tool as region of interest (ROI), and colocalization coefficient within ROI was measured by colocalization tool after the threshold was automatically selected (Costes et al., 2004). Data were statistically analyzed using one-way ANOVA with *post hoc* Tukey–Kramer’s test (Tables 2, 3).

**Table 2:** Colocalization coefficients of Arf4 and Arf5 with markers for intracellular organelles

	GM130	TGN38A	STX12	VAMP4
Arf4	0.43 ± 0.18	0.59 ± 0.09	0.78 ± 0.09	0.67 ± 0.10
	(*n* = 19 cells)	(*n* = 17 cells)	(*n* = 13 cells)	(*n* = 14 cells)
Arf5	0.38 ± 0.13	0.41 ± 0.14	0.64 ± 0.11	0.48 ± 0.14
	(*n* = 18 cells)	(*n* = 24 cells)	(*n* = 19 cells)	(*n* = 22 cells)

**Table 3:** Summary of statistical analyses

Figure number	Panel	Comparison	Data structure: normality	Data structure: homoscedasticity	Type of test	95% confidence interval/Z	*p*-value
Fig. 2	*A, C, E, G*	Colocalization coefficient, Arf4/GM130 vs Arf4/TGN38A vs Arf4/STX12 vs Arf4/VAMP4	Normal distribution, Shapiro–Wilk test; GM130, W = 0.9503, *p *=* *0.4006; TGN38A, W = 0.9475, *p *=* *0.4180; STX12, W = 0.9463, *p *=* *0.5436; VAMP4, W = 0.8853, *p *=* *0.0692	Equal variance, one-way ANOVA, *F*_(3,59)_ = 19.82, *p *=* *0.0001	Tukey–Kramer’s test	Arf4/GM130 vs Arf4/TGN38A, −0.2695 to −0.04074; Arf4/GM130 vs Arf4/STX12, −0.4668 to −0.2202; Arf4/GM130 vs Arf4/VAMP4, −0.3539 to −0.1126; Arf4/TGN38A vs Arf4/STX12, −0.3146 to −0.06218; Arf4/TGN38A vs Arf4/VAMP4, −0.2018–0.04545; Arf4/STX12 vs Arf4/VAMP4, −0.02173–0.2422	Arf4/GM130 vs Arf4/TGN38A, *p *=* *0.0037; Arf4/GM130 vs Arf4/STX12, *p *<* *0.0001; Arf4/GM130 vs Arf4/VAMP4, *p *<* *0.0001; Arf4/TGN38A vs Arf4/STX12, *p *=* *0.0012; Arf4/TGN38A vs Arf4/VAMP4, *p *=* *0.3475; Arf4/STX12 vs Arf4/VAMP4, *p *=* *0.1328
Fig. 2	*B, D, F, H*	Colocalization coefficient, Arf5/GM130 vs Arf5/TGN38A vs Arf5/STX12 vs Arf5/VAMP4	Normal distribution, Shapiro–Wilk test; GM130, W = 0.9246, *p *=* *0.1561; TGN38A, W = 0.9585, *p *=* *0.4085; STX12, W = 0.9636, *p *=* *0.6458; VAMP4, W = 0.9734, *p *=* *0.7885	Equal variance, one-way ANOVA, *F*_(3,79)_ = 12.94, *p *<* *0.0001	Tukey–Kramer’s test	Arf5/GM130 vs Arf5/TGN38A, −0.1459–0.08116; Arf5/GM130 vs Arf5/STX12, −0.3754 to −0.1358; Arf5/GM130 vs Arf5/VAMP4, −0.2166–0.01487; Arf5/TGN38A vs Arf5/STX12, −0.3350 to −0.1114; Arf5/TGN38A vs Arf5/VAMP4, −0.1760–0.03900; Arf4/STX12 vs Arf4/VAMP4, 0.04068–0.2688	Arf5/GM130 vs Arf5/TGN38A, *p *=* *0.8771; Arf5/GM130 vs Arf5/STX12, *p *<* *0.0001; Arf5/GM130 vs Arf5/VAMP4, *p *=* *0.1097; Arf5/TGN38A vs Arf5/STX12, *p *<* *0.0001; Arf5/TGN38A vs Arf5/VAMP4, *p *=* *0.3452; Arf5/STX12 vs Arf5/VAMP4, *p *=* *0.0035
Fig. 3	*A*	Arf4expression, Control vs shArf4#1 vs shArf4#2; Arf5 expression, Control vs shArf4#1 vs shArf4#2	Normal distribution, Shapiro–Wilk test; Arf4, Control, W = 0.9693, *p *=* *0.6634; shArf4#1, W = 0.9060, *p *=* *0.4050; shArf4#2, W = 0.9990, *p *=* *0.9400; Arf5, Control, W = 0.8113, *p *=* *0.1419; shArf4#1, W = 0.8605, *p *=* *0.2689; shArf4#2, W = 0.9127, *p *=* *0.4271	Arf4, equal variance, one-way ANOVA, *F*_(2,6)_ = 10.63, *p = *0.0107; Arf5, equal variance, one-way ANOVA, *F*_(2,6)_ = 0.03027, *p *=* *0.9703	Tukey–Kramer’s test	Arf4, Control vs shArf4#1, 0.2444–1.304; Control vs shArf4#2, 0.01809–1.078; shArf4#1 vs shArf4#2, −0.7562–0.3034; Arf5, Control vs shArf4#1, −0.8078–0.6878; Control vs shArf4#2, −0.7782–0.7174; shArf4#1 vs shArf4#2, −0.7182–0.7774	Arf4, Control vs shArf4#1, *p *=* *0.0099; Control vs shArf4#2, *p *=* *0.0440; shArf4#1 vs shArf4#2, *p *=* *0.9821; Arf5, Control vs shArf4#1, *p *=* *0.9674; Control vs shArf4#2, *p *=* *0.9915; shArf4#1 vs shArf4#2, *p *=* *0.9919
Fig. 3	*B*	Arf4 expression, Control vs shArf5; Arf5 expression, Control vs shArf5	Normal distribution, Shapiro–Wilk test; Arf4, Control, W = 0.9805, *p *=* *0.7325; shArf5, W = 0.9151, *p *=* *0.4353; Arf5, Control, W = 0.8887, *p *=* *0.3503; shArf4#1, W = 0.9465, *p *=* *0.5542	Arf4, equal variance, *F* test, *p *=* *0.0934; Arf5, equal variance, *F* test, *p *=* *0.1375	Unpaired Student’s *t* test	Arf4, Control vs shArf5, −0.8277–2.588; Arf5, Control vs shArf5, −1.367 to −0.2992	Arf4, Control vs shArf5, *p *=* *0.2257; Arf5, Control vs shArf5, *p *=* *0.0123
Fig. 4	*A*	Cell position at E17, Control vs shArf4#1 vs shArf4#2 vs shArf5		Two-way ANOVA, factor for transfected gene, *F*_(3,14)_ = 1.744, *p *=* *0.2039; area factor, *F*_(2,28)_ = 67.04, *p < *0.0001; interaction effect*, F*_(6,28)_ = 18.22*, p *<* *0.0001	Tukey–Kramer’s test	VZ, Control vs shArf4#1, −6.259–13.76; Control vs shArf4#2, −5.319–14.70; Control vs shArf5, −7.425–13.68; shArf4#1 vs shArf4#2, −8.497–10.38; shArf4#1 vs shArf5, −10.63–9.384; shArf4#2 vs shArf5, −11.57–8.444	VZ, Control vs shArf4#1, *p *=* *0.7489; Control vs shArf4#2, *p *=* *0.5971; Control vs shArf5, *p *=* *0.8575; shArf4#1 vs shArf4#2, *p *=* *0.9933; shArf4#1 vs shArf5, *p *=* *0.9983; shArf4#2 vs shArf5, *p *=* *0.9751
						IZ, Control vs shArf4#1, 16.43–36.44; Control vs shArf4#2, 8.766–28.78; Control vs shArf5, −8.700–12.40; shArf4#1 vs shArf4#2, −17.10–1.777; shArf4#1 vs shArf5, −34.59 to −14.58; shArf4#2 vs shArf5, −26.93 to −6.916	IZ, Control vs shArf4#1, *p *<* *0.0001; Control vs shArf4#2, *p *<* *0.0001; Control vs shArf5, *p *=* *0.9654; shArf4#1 vs shArf4#2, *p *=* *0.1480; shArf4#1 vs shArf5, *p *<* *0.0001; shArf4#2 vs shArf5, *p *=* *0.0003
						CP, Control vs shArf4#1, −40.26 to −20.24; Control vs shArf4#2, −33.52 to −13.50; Control vs shArf5, −15.55–5.550; shArf4#1 vs shArf4#2, −2.697–16.18; shArf4#1 vs shArf5, 15.24–35.26; shArf4#2 vs shArf5, 8.501–28.52	CP, Control vs shArf4#1, *p *<* *0.0001; Control vs shArf4#2, *p *<* *0.0001; Control vs shArf5, *p *=* *0.5882; shArf4#1 vs shArf4#2, *p *=* *0.2392; shArf4#1 vs shArf5, *p *<* *0.0001; shArf4#2 vs shArf5, *p *<* *0.0001
Fig. 4	*B*	Cell position at P0, Control vs shArf4#1 vs shArf4#2 vs shArf5		Two-way ANOVA, factor for transfected gene, *F*_(3,14)_ = 9.059, *p *=* *0.0014; area factor, *F*_(3,42)_ = 225.4, *p *<* *0.0001; interaction effect, *F*_(9,42)_ = 47.50, *p *<* *0.0001	Tukey–Kramer’s test	VZ, Control vs shArf4#1, −13.21–6.719; Control vs shArf4#2, −10.88–9.044; Control vs shArf5, −5.375–13.41; shArf4#1 vs shArf4#2, −8.178–12.83; shArf4#1 vs shArf5, −2.699–17.23; shArf4#2 vs shArf5, −5.024–14.90	VZ, Control vs shArf4#1, *p *=* *0.8241; Control vs shArf4#2, *p *=* *0.9948; Control vs shArf5, *p *=* *0.6709; shArf4#1 vs shArf4#2, *p *=* *0.9359; shArf4#1 vs shArf5, *p *=* *0.2272; shArf4#2 vs shArf5, *p *=* *0.5588
						IZ, Control vs shArf4#1, 29.56–49.48; Control vs shArf4#2, −8.144–11.78; Control vs shArf5, −9.095–9.695; shArf4#1 vs shArf4#2, −48.20 to −27.20; shArf4#1 vs shArf5, −49.18 to −29.26; shArf4#2 vs shArf5, −11.48–8.444	IZ, Control vs shArf4#1, *p < *0.0001; Control vs shArf4#2, *p *=* *0.9624; Control vs shArf5, *p *=* *0.9998; shArf4#1 vs shArf4#2, *p < *0.0001; shArf4#1 vs shArf5, *p < *0.0001; shArf4#2 vs shArf5, *p *=* *0.9775
						dCP, Control vs shArf4#1, 19.03–38.96; Control vs shArf4#2, 10.98–30.91; Control vs shArf5, −3.575–15.21; shArf4#1 vs shArf4#2, −18.55–2.453; shArf4#1 vs shArf5, −33.14 to −13.21; shArf4#2 vs shArf5, −25.09 to −5.161	dCP, Control vs shArf4#1, *p *<* *0.0001; Control vs shArf4#2, *p *<* *0.0001; Control vs shArf5, *p *=* *0.3648; shArf4#1 vs shArf4#2, *p = *0.1896; shArf4#1 vs shArf5, *p *<* *0.0001; shArf4#2 vs shArf5, *p *=* *0.0010
						uCP, Control vs shArf4#1, −75.19 to −55.27; Control vs shArf4#2, −36.29 to −16.37; Control vs shArf5, −19.51 to −0.7255; shArf4#1 vs shArf4#2, 28.40–49.40; shArf4#1 vs shArf5, 45.15–65.07; shArf4#2 vs shArf5, 6.246–26.17	uCP, Control vs shArf4#1, *p *<* *0.0001; Control vs shArf4#2, *p *<* *0.0001; Control vs shArf5, *p *=* *0.0300; shArf4#1 vs shArf4#2, *p *<* *0.0001; shArf4#1 vs shArf5, *p *<* *0.0001; shArf4#2 vs shArf5, *p *=* *0.0004
Fig. 4	*C*	Cell position at P0, Rescue, shArf4#1 vs shArf4#1/CAG-Arf4 vs shArf4#1/CAG-Arf5		Two-way ANOVA, factor for transfected gene, *F*_(2,9)_ = 0.6429, *p* = 0.5483; area factor, *F*_(3,27)_ = 23.11, *p* < 0.0001; interaction effect, *F*_(6,27)_ = 5.491, *p* = 0.0008	Tukey–Kramer’s test	VZ, shArf4#1 vs shArf4#1/CAG-Arf4, −11.95–18.80; shArf4#1 vs shArf4#1/CAG-Arf5, −15.55–15.20; shArf4#1/CAG-Arf4 vs shArf4#1/CAG-Arf5, −18.97–11.77	VZ, shArf4#1 vs shArf4#1/CAG-Arf4, *p *=* *0.8499; shArf4#1 vs shArf4#1/CAG-Arf5, *p *=* *0.9996; shArf4#1/CAG-Arf4 vs shArf4#1/CAG-Arf5, *p *=* *0.8356
						IZ, shArf4#1 vs shArf4#1/CAG-Arf4, −42.65 to −11.90; shArf4#1 vs shArf4#1/CAG-Arf5, −15.47–15.27; shArf4#1/CAG-Arf4 vs shArf4#1/CAG-Arf5, 11.80–42.55	IZ, shArf4#1 vs shArf4#1/CAG-Arf4, *p *=* *0.0003; shArf4#1 vs shArf4#1/CAG-Arf5, *p *=* *0.9999; shArf4#1/CAG-Arf4 vs shArf4#1/CAG-Arf5, *p *=* *0.0003
						dCP, shArf4#1 vs shArf4#1/CAG-Arf4, −15.25–15.50; shArf4#1 vs shArf4#1/CAG-Arf5, −15.60–15.15; shArf4#1/CAG-Arf4 vs shArf4#1/CAG-Arf5, −15.72–15.02	dCP, shArf4#1 vs shArf4#1/CAG-Arf4, *p *=* *0.9998; shArf4#1 vs shArf4#1/CAG-Arf5, *p *=* *0.9993; shArf4#1/CAG-Arf4 vs shArf4#1/CAG-Arf5, *p *=* *0.9983
						uCP, shArf4#1 vs shArf4#1/CAG-Arf4, 8.303–39.05; shArf4#1 vs shArf4#1/CAG-Arf5, −14.90–15.85; shArf4#1/CAG-Arf4 vs shArf4#1/CAG-Arf5, −38.57 to −7.828	uCP, shArf4#1 vs shArf4#1/CAG-Arf4, *p *=* *0.0017; shArf4#1 vs shArf4#1/CAG-Arf5, *p *=* *0.9969; shArf4#1/CAG-Arf4 vs shArf4#1/CAG-Arf5, *p *=* *0.0021
Fig. 4	*C*	Cell position at P0, Rescue, shArf4#1 vs shArf4#1/CAG-Arf4 vs shArf4#1/CAG-Arf5		Two-way ANOVA, factor for transfected gene, *F*_(2,9)_ = 0.6429, *p* = 0.5483; area factor, *F*_(3,27)_ = 23.11, *p* < 0.0001; interaction effect, *F*_(6,27)_ = 5.491, *p* = 0.0008	Tukey–Kramer’s test	VZ, shArf4#1 vs shArf4#1/CAG-Arf4, −11.95–18.80; shArf4#1 vs shArf4#1/CAG-Arf5, −15.55–15.20; shArf4#1/CAG-Arf4 vs shArf4#1/CAG-Arf5, −18.97–11.77	VZ, shArf4#1 vs shArf4#1/CAG-Arf4, *p *=* *0.8499; shArf4#1 vs shArf4#1/CAG-Arf5, *p *=* *0.9996; shArf4#1/CAG-Arf4 vs shArf4#1/CAG-Arf5, *p *=* *0.8356
						IZ, shArf4#1 vs shArf4#1/CAG-Arf4, −42.65 to −11.90; shArf4#1 vs shArf4#1/CAG-Arf5, −15.47–15.27; shArf4#1/CAG-Arf4 vs shArf4#1/CAG-Arf5, 11.80–42.55	IZ, shArf4#1 vs shArf4#1/CAG-Arf4, *p *=* *0.0003; shArf4#1 vs shArf4#1/CAG-Arf5, *p *=* *0.9999; shArf4#1/CAG-Arf4 vs shArf4#1/CAG-Arf5, *p *=* *0.0003
						dCP, shArf4#1 vs shArf4#1/CAG-Arf4, −15.25–15.50; shArf4#1 vs shArf4#1/CAG-Arf5, −15.60–15.15; shArf4#1/CAG-Arf4 vs shArf4#1/CAG-Arf5, −15.72–15.02	dCP, shArf4#1 vs shArf4#1/CAG-Arf4, *p *=* *0.9998; shArf4#1 vs shArf4#1/CAG-Arf5, *p *=* *0.9993; shArf4#1/CAG-Arf4 vs shArf4#1/CAG-Arf5, *p *=* *0.9983
						uCP, shArf4#1 vs shArf4#1/CAG-Arf4, 8.303–39.05; shArf4#1 vs shArf4#1/CAG-Arf5, −14.90–15.85; shArf4#1/CAG-Arf4 vs shArf4#1/CAG-Arf5, −38.57 to −7.828	uCP, shArf4#1 vs shArf4#1/CAG-Arf4, *p *=* *0.0017; shArf4#1 vs shArf4#1/CAG-Arf5, *p *=* *0.9969; shArf4#1/CAG-Arf4 vs shArf4#1/CAG-Arf5, *p *=* *0.0021
Fig. 5	*A*	Arf4 expression, Control vs shArf4#1	Non-normal distribution, Shapiro–Wilk test; control, W = 0.9654, *p *=* *0.3642; shArf4#1, W = 0.9340, *p *=* *0.0299		Mann–Whitney *U test*	−0.6000 to −0.4000	*p* < 0.0001
Fig. 5	*B*	Arf5 expression, Control vs shArf5	Non-normal distribution, Shapiro–Wilk test; control, W = 0.1509, *p *>* *0.1000; shArf5, W = 0.2560, *p *=* *0.0009		Mann–Whitney *U test*	−0.9000 to −0.5000	*p* < 0.0001
Fig. 5	*C*	Cell position at E15, Control vs shArf4#1 vs shArf5		Two-way ANOVA, factor for transfected gene, *F*_(2,12)_ = 2.667, *p *=* *0.1101; area factor, *F*_(1.735,20.82)_ = 94.17, *p < *0.0001; interaction effect*, F*_(4,24)_ = 1.292, *p = *0.3009	Tukey–Kramer’s test	VZ, Control vs shArf4#1, −9.017–16.36; Control vs shArf5, −8.638–18.89; shArf4#1 vs shArf5, −10.93–13.83	VZ, Control vs shArf4#1, *p *=* *0.6921; Control vs shArf5, *p *=* *0.5604; shArf4#1 vs shArf5, *p *=* *0.9393
						SVZ, Control vs shArf4#1, −11.89–7.750; Control vs shArf5, −21.18–4.906; shArf4#1 vs shArf5, −18.93–6.807	SVZ, Control vs shArf4#1, *p *=* *0.8222; Control vs shArf5, *p *=* *0.2274; shArf4#1 vs shArf5, *p *=* *0.3934
						IZ, Control vs shArf4#1, −5.604–2.400; Control vs shArf5, −9.477–15.49; shArf4#1 vs shArf5, −8.015–17.23	IZ, Control vs shArf4#1, *p *=* *0.5028; Control vs shArf5, *p *=* *0.7215; shArf4#1 vs shArf5, *p *=* *0.4841
Fig. 5	*D*	BrdU+ cells, Control vs shArf4#1 vs shArf5	Normal distribution, Shapiro–Wilk test; Control, W = 0.9207, *p *=* *0.5342; shArf4#1, W = 0.9454, *p *=* *0.7040; shArf5, W = 0.9257, *p *=* *0.5674	Equal variance, one-way ANOVA, *F*_(2,12)_ = 2.029, *p *=* *0.1742	Tukey–Kramer’s test	Control vs shArf4#1, −4.013–10.03; Control vs shArf5, −1.737–12.30; shArf4#1 vs shArf5, −4.745–9.297	Control vs shArf4#1, *p *=* *0.5074; Control vs shArf5, *p *=* *0.1526; shArf4#1 vs shArf5, *p *=* *0.6715
Fig. 5	*E*	PHH3+ cells, Control vs shArf4#1 vs shArf5	Normal distribution, Shapiro–Wilk test; Control, W = 0.9866, *p *=* *0.9664; shArf4#1, W = 0.8926, *p *=* *0.3951; shArf5, W = 0.8718, *p *=* *0.3007	Equal variance, one-way ANOVA, *F*_(2,9)_ = 0.9211, *p *=* *0.4326	Tukey–Kramer’s test	Control vs shArf4#1, −0.08036–0.05991; Control vs shArf5, −0.04857–0.1041; shArf4#1 vs shArf5, −0.04185–0.1179	Control vs shArf4#1, *p *=* *0.9136; Control vs shArf5, *p *=* *0.586; shArf4#1 vs shArf5, *p *=* *0.4155
Fig. 5	*F*	Orientation of GM130, Control vs shArf4#1 vs shArf5	Normal distribution, Shapiro–Wilk test; Control, W = 0.9692, *p *=* *0.6632; shArf4#1, W = 0.9959, *p *=* *0.8776; shArf5, W = 0.9750, *p *=* *0.6967	Equal variance, one-way ANOVA, *F*_(2,6)_ = 19.27, *p *=* *0.0024	Tukey–Kramer’s test	Control vs shArf4#1, 10.86–38.54; Control vs shArf5, −12.91–14.77; shArf4#1 vs shArf5, −37.61 to −9.928	Control vs shArf4#1, *p *=* *0.0037; Control vs shArf5, *p *=* *0.9768; shArf4#1 vs shArf5, *p *=* *0.0045
Fig. 5	*H*	Cell morphology at E16, Control vs shArf4#1 vs shArf5		Two-way ANOVA, factor for transfected gene, *F*_(2,24)_ = 0.002203, *p *=* *0.9978; factor for morphology, *F*_(3,24)_ = 1080, *p* < 0.0001; interaction effect, *F*_(4,24)_ = 1.292, *p* = 0.3009	Tukey–Kramer’s test	Bipolar; Control vs shArf4#1, −0.1124–0.03241; Control vs shArf5, −0.07574–0.06908; shArf4#1 vs shArf5, −0.03574–0.1091	Bipolar; Control vs shArf4#1, *p *=* *0.3671; Control vs shArf5, *p *=* *0.9927; shArf4#1 vs shArf5, *p *=* *0.4283
						Rounding; Control vs shArf4#1, −0.06574–0.07908; Control vs shArf5, −0.08241–0.06241; shArf4#1 vs shArf5, −0.08908–0.05574	Rounding; Control vs shArf4#1, *p *=* *0.9713; Control vs shArf5, *p *=* *0.9367; shArf4#1 vs shArf5, *p *=* *0.8347
						Multipolar; Control vs shArf4#1, −0.03241–0.1124; Control vs shArf5, −0.04908–0.09574; shArf4#1 vs shArf5, −0.08908–0.05574	Multipolar; Control vs shArf4#1, *p *=* *0.3671; Control vs shArf5, *p *=* *0.7038; shArf4#1 vs shArf5, *p *=* *0.8347
						Unknown; Control vs shArf4#1, −0.08241–0.06241; Control vs shArf5, −0.08241–0.06241; shArf4#1 vs shArf5, −0.07241–0.07241	Unknown; Control vs shArf4#1, *p *=* *0.9367; Control vs shArf5, *p *=* *0.9367; shArf4#1 vs shArf5, *p *>* *0.9999
Fig. 5	*J*	Cell morphology at E17, Control vs shArf4#1 vs shArf5		Two-way ANOVA, factor for transfected gene, *F*_(2,24)_ = 0.002203, *p *=* *0.9978; factor for morphology, *F*_(3,24)_ = 1080, *p *<* *0.0001; interaction effect, *F*_(6,24)_ = 0.7922, *p *=* *0.5850	Tukey–Kramer’s test	Bipolar; Control vs shArf4#1, −0.1124–0.03241; Control vs shArf5, −0.07574–0.06908; shArf4#1 vs shArf5, −0.1124–0.03241	Bipolar; Control vs shArf4#1, *p *=* *0.8347; Control vs shArf5, *p *=* *0.9367; shArf4#1 vs shArf5, *p *=* *0.9713
						Rounding; Control vs shArf4#1, −0.08908–0.05574; Control vs shArf5, −0.08241–0.06241; shArf4#1 vs shArf5, −0.06574–0.07908	Rounding; Control vs shArf4#1, *p *=* *0.8347; Control vs shArf5, *p *=* *0.7038; shArf4#1 vs shArf5, *p *=* *0.3671
						Multipolar; Control vs shArf4#1, −0.08908–0.05574; Control vs shArf5, −0.04908–0.09574; shArf4#1 vs shArf5, −0.03241–0.1124	Multipolar; Control vs shArf4#1, *p *=* *0.3671; Control vs shArf5, *p *=* *0.7038; shArf4#1 vs shArf5, *p *=* *0.8347
						Unknown; Control vs shArf4#1, −0.07241–0.07241; Control vs shArf5, −0.08241–0.06241; shArf4#1 vs shArf5, −0.08241–0.06241	Unknown; Control vs shArf4#1, *p *>* *0.9999; Control vs shArf5, *p *=* *0.9367; shArf4#1 vs shArf5, *p *=* *0.9367
Fig. 5	*K*	Number of cell process, Control vs shArf4#1 vs shArf5		Two-way ANOVA, factor for transfected gene, *F*_(2,28)_ = 0.000, *p *>* *0.9999; factor for morphology, *F*_(3,28)_ = 23.54, *p *<* *0.0001; interaction effect, *F*_(6,28)_ = 16.40, *p *<* *0.0001	Tukey–Kramer’s test	0–3; Control vs shArf4#1, −21.63–8.378; Control vs shArf5, −14.96–15.04; shArf4#1 vs shArf5, −9.372–22.71	0–3; Control vs shArf4#1, *p *=* *0.5264; Control vs shArf5, *p *>* *0.9999; shArf4#1 vs shArf5, *p *=* *0.5655
						4–6; Control vs shArf4#1, −47.67 to −17.66; Control vs shArf5, −10.50–19.50; shArf4#1 vs shArf5, 21.13–53.21	4–6; Control vs shArf4#1, *p *<* *0.0001 Control vs shArf5, *p *=* *0.7408; shArf4#1 vs shArf5, *p *<* *0.0001
						7–9; Control vs shArf4#1, −11.43–18.58; Control vs shArf5, −9.761–20.24; shArf4#1 vs shArf5, −14.37–17.71	7–9; Control vs shArf4#1, *p *=* *0.8268; Control vs shArf5, *p *=* *0.6667; shArf4#1 vs shArf5, *p *=* *0.9643
						>10; Control vs shArf4#1, 20.71–50.72; Control vs shArf5, −24.79–5.219; shArf4#1 vs shArf5, −61.54 to −29.46	>10; Control vs shArf4#1, *p *<* *0.0001; Control vs shArf5, *p *=* *0.2567; shArf4#1 vs shArf5, *p *<* *0.0001
Fig. 6	*B*	Timelaps, E17, IZ, Control vs shArf4#1	Non-normal distribution, Shapiro–Wilk test; control, W = 0.9716, *p *=* *0.5849; shArf4#1, W = 0.8983, *p *=* *0.0077		Mann–Whitney *U test*	−5.400 to −3.200	*p *<* *0.0001
Fig. 6	*D*	Timelaps, E17, CP, Control vs shArf4#1	Non-normal distribution, Shapiro–Wilk test; control, W = 0.9411, *p *=* *0.0977; shArf4#1, W = 0.9299, *p *=* *0.0489		Mann–Whitney *U test*	−8.740 to −4.500	*p *<* *0.0001
Fig. 8	*A*	GM130; Control vs shArf4#1 vs shArf5	Non-normal distribution, Shapiro–Wilk test; control, W = 0.9809, *p *=* *0.2083; shArf4#1, W = 0.9885, *p *=* *0.7210; shArf5, W = 0.8227, *p *<* *0.0001	Unequal variance, Kruskal-Wallis test, *p *<* *0.0001	Dunn’s multiple comparison test		Control vs shArf4#1, *p *<* *0.0001; Control vs shArf5, *p *=* *0.0006
Fig. 8	*B*	STX16; Control vs shArf4#1 vs shArf5	Non-normal distribution, Shapiro–Wilk test; control, W = 0.9809, *p *=* *0.2083; shArf4#1, W = 0.9885, *p *=* *0.7210; shArf5, W = 0.8227, *p *<* *0.0001	Unequal variance, Kruskal-Wallis test, *p *<* *0.0001	Dunn’s multiple comparison test		Control vs shArf4#1, *p *<* *0.0001; Control vs shArf5, *p *=* *0.0068
Fig. 8	*C*	VAMP4; Control vs shArf4#1 vs shArf5	Non-normal distribution, Shapiro–Wilk test; control, W = 0.8348, *p *<* *0.0001; shArf4#1, W = 0.8728, *p *=* *0.0429; shArf5, W = 0.9070, *p *<* *0.0001	Unequal variance, Kruskal-Wallis test, *p *<* *0.0001	Dunn’s multiple comparison test		Control vs shArf4#1, *p *<* *0.0001; Control vs shArf5, *p *=* *0.4254
Fig. 8	*D*	STX12; Control vs shArf4#1 vs shArf5	Non-normal distribution, Shapiro–Wilk test; control, W = 0.7465, *p *<* *0.0001; shArf4#1, W = 0.7369, *p *<* *0.0001; shArf5, W = 0.8304, *p *<* *0.0001	Unequal variance, Kruskal-Wallis test, *p *<* *0.0001	Dunn’s multiple comparison test		Control vs shArf4#1, *p *<* *0.0001; Control vs shArf5, *p *>* *0.9999
Fig. 8	*E*	Rab11; Control vs shArf4#1 vs shArf5	Non-normal distribution, Shapiro–Wilk test; control, W = 0.8978, *p *<* *0.0001; shArf4#1, W = 0.7270, *p *<* *0.0001; shArf5, W = 0.8342, *p *<* *0.0001	Unequal variance, Kruskal-Wallis test, *p *<* *0.0001	Dunn’s multiple comparison test		Control vs shArf4#1, *p *<* *0.0001; Control vs shArf5, *p *=* *0.8966
Fig. 8	*F*	TGN38A; Control vs shArf4#1 vs shArf5	Non-normal distribution, Shapiro–Wilk test; control, W = 0.9113, *p *<* *0.0001; shArf4#1, W = 0.8742, *p *<* *0.0001; shArf5, W = 0.9744, *p *=* *0.1315	Unequal variance, Kruskal-Wallis test, *p *<* *0.0001	Dunn’s multiple comparison test		Control vs shArf4#1, *p *<* *0.0001; Control vs shArf5, *p *<* *0.0001
Fig. 8	*G*	EEA1; Control vs shArf4#1 vs shArf5	Non-normal distribution, Shapiro–Wilk test; control, W = 0.8505, *p *<* *0.0001; shArf4#1, W = 0.7961, *p *<* *0.0001; shArf5, W = 0.8918, *p *<* *0.0001	Unequal variance, Kruskal-Wallis test, *p *=* *0.217	Dunn’s multiple comparison test		Control vs shArf4#1, *p *=* *0.5118; Control vs shArf5, *p *=* *0.1680
Fig. 8	*A*	Fluorescence intensity, N-cad, Control vs shArf4#1 vs shArf5	Non-normal distribution, Shapiro–Wilk test; control, W = 0.9391, *p *<* *0.0001; shArf4#1, W = 0.9442, *p *<* *0.0001; shArf5, W = 0.9377, *p *=* *0.0001	Unequal variance, Kruskal-Wallis test, *p *<* *0.0001	Dunn’s multiple comparison test		Control vs shArf4#1, *p *<* *0.0001; Control vs shArf5, *p *=* *0.4333
Fig. 8	*B*	Colocalization coefficient, shArf4#1, STX16/N-cad vs TGN38A/N-cad, STX16/N-cad vs VAMP4/N-cad, TGN38A/N-cad vs VAMP4/N-cad	Non-normal distribution, Shapiro–Wilk test; STX16/N-cad, W = 0.8855, *p *=* *0.0023; TGN38A/N-cad, W = 0.5834, *p *<* *0.0001; VAMP4/N-cad, W = 0.9287, *p *=* *0.0256	Unequal variance, Kruskal-Wallis test, *p* < 0.0001	Dunn’s multiple comparison test		STX16/N-cad vs TGN38A/N-cad, *p *<* *0.0001; STX16/N-cad vs VAMP4/N-cad, *p *=* *0.5824; TGN38A/N-cad vs VAMP4/N-cad, *p *<* *0.0001
Fig. 8	*C*	Contact index, Control vs shArf4#1	Non-normal distribution, Shapiro–Wilk test; control, W = 0.9538, *p *=* *0.0824; shArf4#1, W = 0.9081, *p *=* *0.0029		Mann–Whitney *U test*	−0.3000 to −0.1000	*p = *0.0004
Fig. 9	*D*	Length of leading process, Control vs shArf4#1	Non-normal distribution, Shapiro–Wilk test; control, W = 0.9232, *p *<* *0.0001; shArf4#1, W = 0.9551, *p *<* *0.0001		Mann–Whitney *U test*	−3.900–0.7000	*p *=* *0.1696
Fig. 9	*E*	Cell position at P0, Rescue, shArf4#1 vs shArf4#1/CAG-Ncad		Two-way ANOVA, factor for transfected gene, *F*_(1,7)_ = 2.032, *p *=* *0.1970; Area factor, *F*_(3,21)_ = 27.93, *p *<* *0.0001; interaction effect, *F*_(3,21)_ = 8.333, *p *=* *0.0008	Bonferroni’s multiple comparisons test	VZ, −13.53–9.280; IZ, −30.47 to −7.660; dCP, −3.520–19.29; uCP, 1.945–24.75	VZ, *p *>* *0.9999; IZ, *p *=* *0.0005; dCP, *p *=* *0.3022; uCP, *p *=* *0.0165

The number of EGFP-labeled or mCherry-labeled transfected cells in each cortical zone was counted using Fiji software. Each cortical zone was identified by the following criteria: In E15 brain sections, the VZ and SVZ were visualized by the immunoreactivity for Sox2 and the VZ and SVZ were identified by nuclear shapes, with DAPI staining. In E17 brain sections, the VZ, IZ, and CP were identified by the nuclear density with DAPI staining. In neonatal brain sections, upper cortical plate (uCP), and deep cortical plate (dCP) were identified by the combination of the immunoreactivity for Cux1, a marker for upper cortical layers, and nuclear density by DAPI staining, and the IZ and VZ were further distinguished by DAPI staining as low-cell-density and high-cell-density zones, respectively. Data in each experimental condition were taken using two consecutive sections from four to five individual embryos or neonates in two pregnant mice per group. The percentage of EGFP-labeled or mCherry-labeled cells in each cortical zone was compared with that in the corresponding zone in control animals transfected with control shRNA or indicated plasmids. Data were statistically analyzed using two-way ANOVA with the *post hoc* Tukey–Kramer’s test (Table 3), or Bonferroni test ( Table 3).

The number of cell processes was counted by marking cell processes extending from the cell body and a leading process of EGFP-labeled transfected cells in the upper IZ at E17 using counter tool of Fiji software with sequential images. Data were statistically analyzed using two-way ANOVA with the *post hoc* Tukey–Kramer’s test (Table 3).

The analyses of organelle morphology were performed using Fiji software as follows: EGFP-labeled transfected cells in the IZ were selected by outlining their contours using polygon selection tools. The channel image for an organelle marker was duplicated into a new window, and immunoreactive puncta were extracted by setting thresholds to obtain data using the command “analyze particle.” The ratios of total areas of GM130-immunoreactive, TGN38A-immunoreactive, early endosome antigen 1 (EEA1)-immunoreactive, STX12-immunoreactive, Rab11-immunoreactive, STX16-immunoreactive, or VAMP4-immunoreactive puncta to those of cell soma were statistically analyzed using Kruskal–Wallis test followed by Dunn’s multiple comparison test (Table 3).

The fluorescence intensity for N-cadherin in the cell body was obtained by subtracting that in the nucleus in transfected cells, and normalized by that of control shRNA-transfected cells. Data were statistically analyzed using Kruskal–Wallis test followed by Dunn’s multiple comparison test (Table 3).

The contact index was defined as the ratio of the contact length of an EGFP-positive migrating neuron with BLBP-immunoreactive radial glial fibers to the total length of its cell body and leading process observed on a single image, as indicated in Figure 9*C*. Data were analyzed statistically using Mann–Whitney *U* test (Table 3).

The length of a leading process was measured by tracing a leading process of EGFP-labeled transfected cells in the IZ at E17 from the distal tip to the proximal base at the cell body using segmental line tools of ZEN software with stacked images. Data were analyzed statistically using Mann–Whitney *U* test (Table 3). Statistical analyses in this study were performed using the GraphPad Prism9.0 for Macintosh (GraphPad Software; RRID: SCR_002798).

## Results

### Class II Arfs exhibit overlapping but distinct expression in the developing cerebral cortex

**Figure 1.**
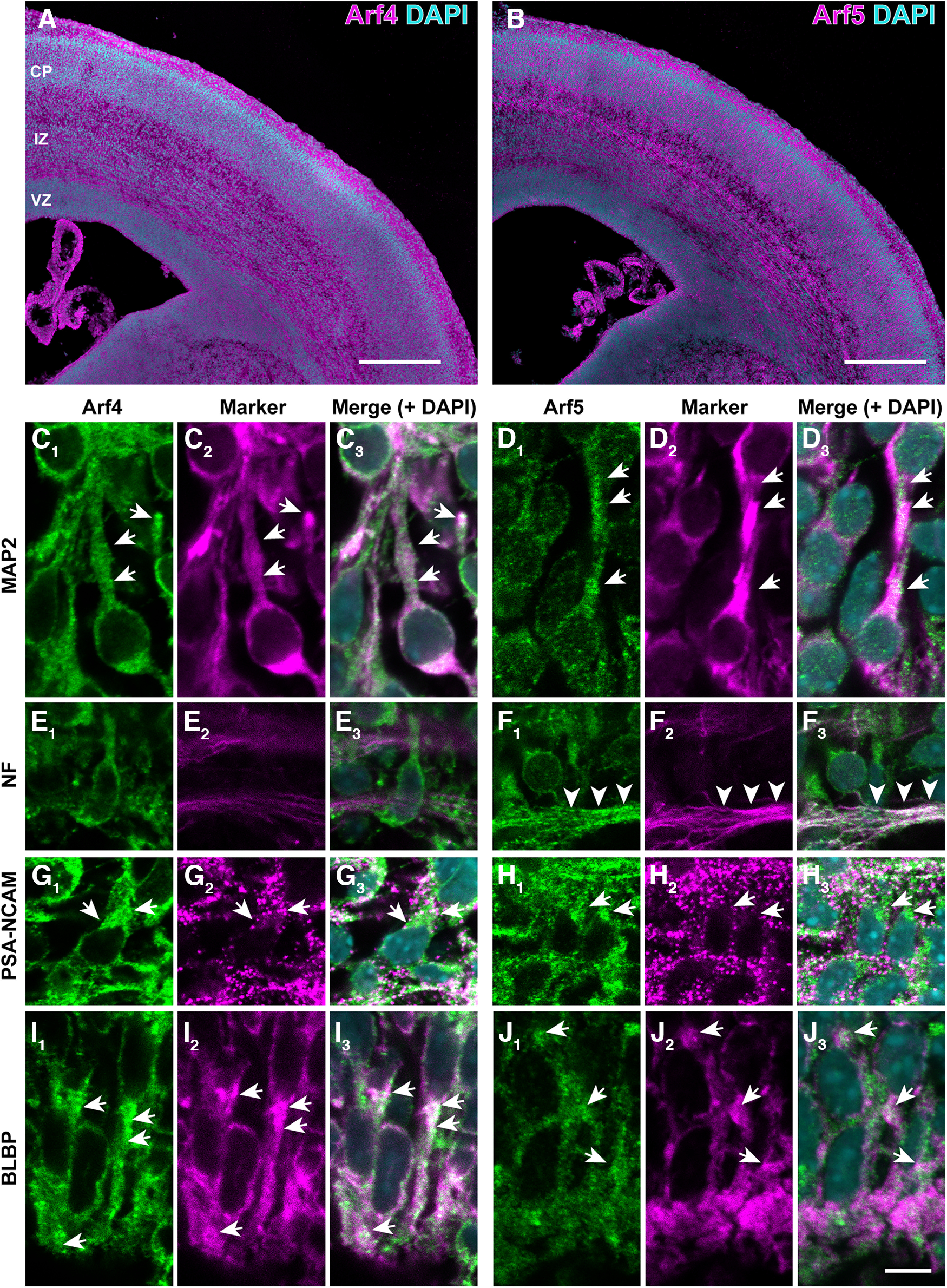
Cellular expression of Arf4 and Arf5 in the developing cerebral cortex. ***A***, ***B***, Immunofluorescence staining of coronal sections of the cerebral cortex at E17 with antibodies against Arf4 (***A***) and Arf5 (***B***). Note the expression of Arf4 and Arf5 proteins throughout cortical zones, including ventricular zone (VZ), intermediate zone (IZ), and cortical plate (CP). ***C–J***, Double immunofluorescence staining of the cerebral cortex at E17 with antibodies against Arf4 (***C*_1_**, ***E*_1_**, ***G*_1_**, ***I*_1_**) or Arf5 (***D*_1_**, ***F*_1_**, ***H*_1_**, ***J*_1_**) and MAP2 (***C*_2_**, ***D*_2_**), neurofilament 165 (NF; ***E*_2_**, ***F*_2_**), PSA-NCAM (***G*_2_**, ***H*_2_**), or BLBP (***I*_2_**, ***J*_2_**). Arrows indicate the expression of Arf4 and Arf5 in MAP2-positive postmigratory neurons in the CP (***C***, ***D***), PSA-NCAM-positive migrating neurons in the IZ (***G***, ***H***), and BLBP-positive radial glia in the VZ (***I***, ***J***). Arrowheads in ***F*** indicate intense immunoreactivity for Arf5, but not Arf4, in NF-positive axons in the IZ. Scale bars: 400 μm in ***A*** and ***B***, and 10 μm in ***J*_3_**.

A previous *in situ* hybridization study has shown that Arf4 and Arf5 mRNAs are substantially expressed in the developing rat brain (Suzuki et al., 2001). To examine the expression of Arf4 and Arf5 proteins in developing cerebral cortices, we performed immunohistological analyses of the mouse cerebral cortex using specific antibodies against Arf4 and Arf5 (Hosoi et al., 2019). In the dorsal pallium at E17 (Fig. 1*A*,*B*), both proteins were expressed throughout the cerebral zones, including the VZ, IZ, and CP (Fig. 1*A*,*B*). We further performed double immunofluorescence using antibodies against Class II Arfs and microtubule-associated protein-2 (MAP2) for postmigratory neurons, neurofilament (NF) 165 for axons, the polysialylated neural adhesion molecule (PSA-NCAM) for immature neurons, and the brain lipid-binding protein (BLBP) for radial glia. Both Arf4 and Arf5 were expressed prominently in cell bodies and proximal processes of MAP2-positive postmigratory neurons in the CP (Fig. 1*C*,*D*) and PSA-NCAM-positive migrating neurons in the IZ (Fig. 1*G*,*H*), and BLBP-positive radial glia in the VZ (Fig. 1*I*,*J*). In the IZ, intense immunofluorescence for Arf5, but not for Arf4, was observed in the axon bundle labeled by NF165, presumably corresponding to developing fibers projecting to subcortical regions (Fig. 1*F*, arrowheads). These results suggest that both Arf4 and Arf5 are widely expressed in the developing cerebral cortex.

**Figure 2.**
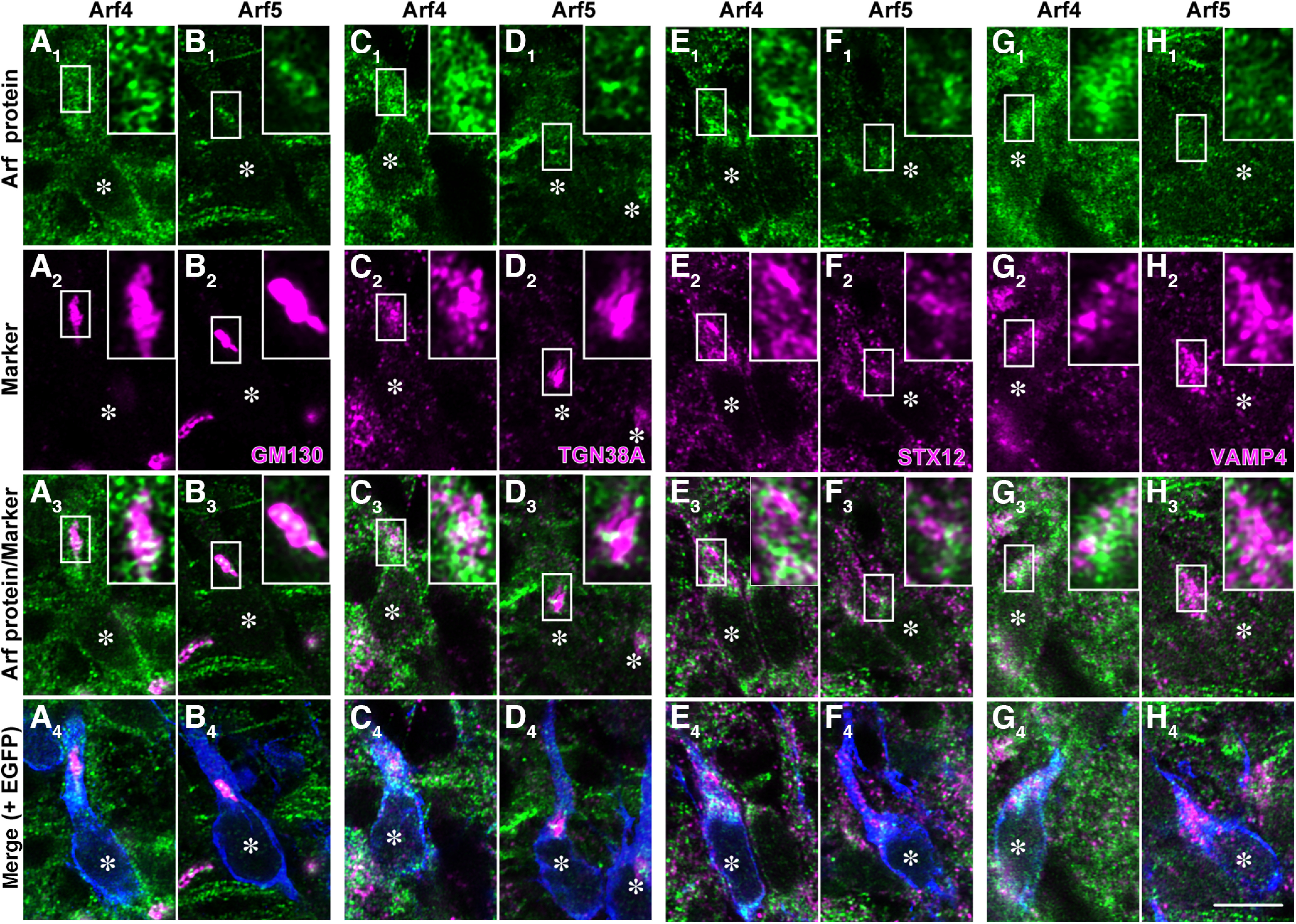
Subcellular localization of Arf4 and Arf5 in migrating neurons. Representative micrographs showing the colocalization of Arf4 (***A*_1_**, ***C*_1_**, ***E*_1_**, ***G*_1_**) or Arf5 (***B*_1_**, ***D*_1_**, ***F*_1_**, ***H*_1_**) with GM130 (for *cis*-Golgi; ***A*_2_**, ***B*_2_**), TGN38A (for the TGN; ***C*_2_**, ***D*_2_**), STX12 (for recycling endosomes; ***E*_2_**, ***F*_2_**), or VAMP4 (for retrograde transporting vesicles; ***G*_2_**, ***H*_2_**) in migrating neurons in the IZ at E17. Coronal sections of the E17 cerebral cortex that had been electroporated with pCAGGS-EGFP at E14 were subjected to immunofluorescence staining with antibodies against Arf4 or Arf5 (green), indicated markers (magenta), and EGFP (blue). Merged images (***A*_4_–*H*_4_**) include EGFP immunofluorescence (blue) in transfected neurons. Asterisks indicate the nuclei of EGFP-labeled transfected migrating neurons. Insets show the high-magnification views of boxed areas. Scale bar: 10 μm in ***H*_4_**.

To further examine the subcellular localization of Arf4 and Arf5, migrating neurons in the IZ at E17 were visualized by expressing EGFP using IUE at E14 and subjected to immunofluorescence staining with antibodies against Arf4 or Arf5 and various organelle markers. Both Arf4 and Arf5 appeared as numerous puncta throughout the cytoplasm (Fig. 2*A–H*), which overlapped partially with GM130 (for *cis*-Golgi; Nakamura et al., 1995; Fig. 2*A*,*B*), TGN38A [for the *trans*-Golgi network (TGN); Luzio et al., 1990; Ibuchi et al., 2020; Fig. 2*C*,*D*], syntaxin 12 (STX12; for recycling endosomes; Prekeris et al., 1998; Hara et al., 2013; Fig. 2*E*,*F*), and VAMP4 (for retrograde transport vesicles; Mallard et al., 2002; Fig. 2*G*,*H*). Quantification of the colocalization coefficient revealed that both Arf4 and Arf5 localized to various organelles with subtly different preferences (Tables 2, 3), suggesting that Class II Arfs mediate various steps of membrane trafficking in migrating neurons.

### Arf4 regulates radial migration during cortical development

**Figure 3.**
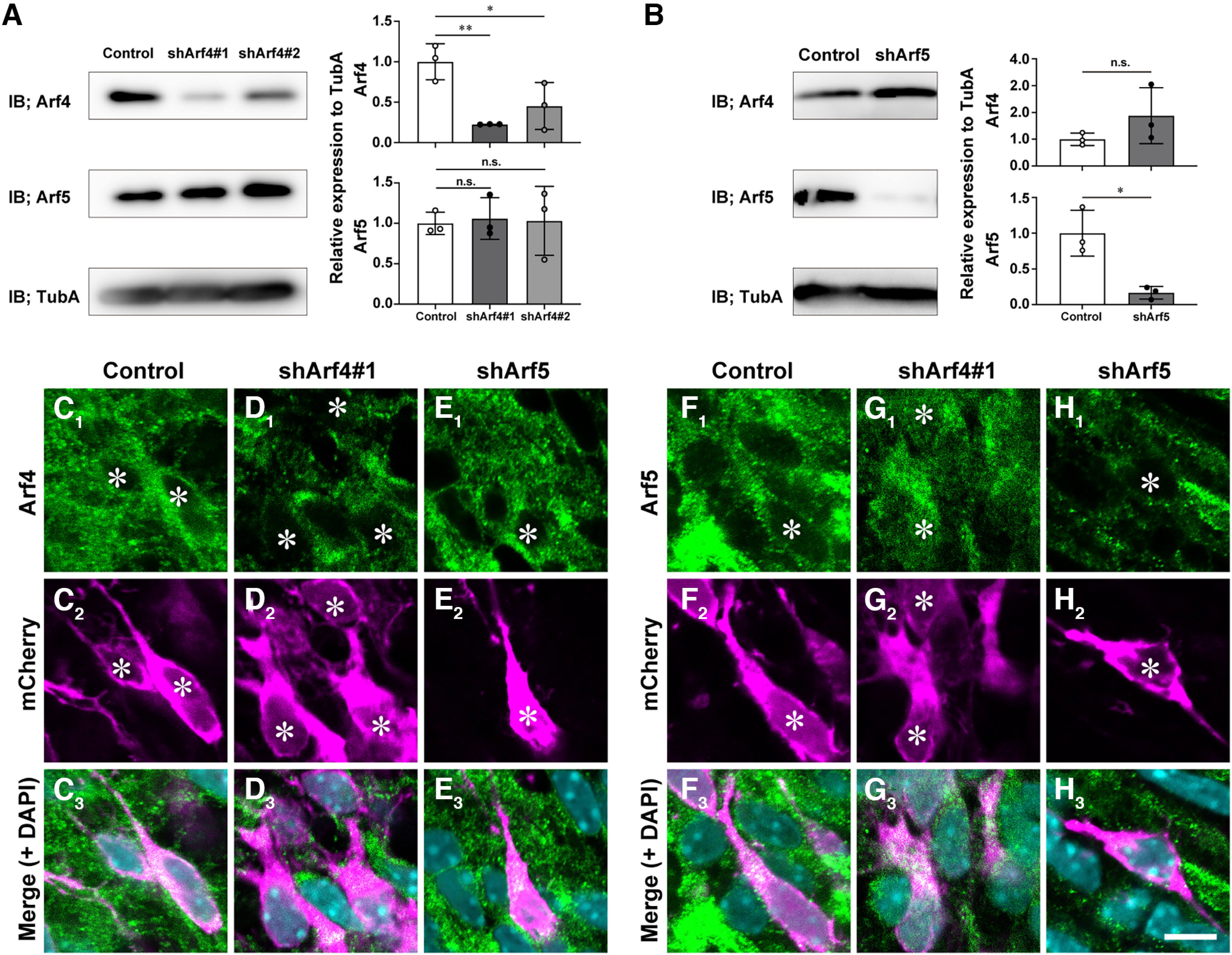
Characterization of shRNAs against Arf4 and Arf5. ***A***, ***B***, Immunoblotting. Primary cortical neurons were transfected with control or indicated shRNA vector and subjected to immunoblotting (IB) with antibodies against Arf4, Arf5, and α-tubulin (TubA). The graphs show the relative expression of Arf4 and Arf5 to that of TubA. ***C*–*H***, Representative immunofluorescence images showing the effect of control shRNA (***C***, ***F***), shArf4#1 (***D***, ***G***), or shArf5 (***E***, ***H***) on endogenous expression of Arf4 (***C*_1_–*E*_1_**) and Arf5 (***F*_1_–*H*_1_**) in migrating neurons in the IZ. Sections of the E17 cerebral cortices that had been electroporated with indicated shRNAs and mCherry at E14 were subjected to double immunofluorescence with antibodies against Arf4 (***C*_1_–*E*_1_**) or Arf5 (***F*_1_–*H*_1_**) and mCherry (***C*_2_–*H*_2_**). Note the decrease in endogenous expression of the respective Arf protein by transfecting with shArf4#1 without compensatory upregulation of the other. Asterisks indicate the nuclei of transfected cells. Data were presented as mean ± SD and statistically analyzed by one-way ANOVA with *post hoc* Tukey–Kramer’s test (***p <* *0.0005) in ***A*** or unpaired Student’s *t* tests in ***B*** (*p < 0.05, n.s., not significant). Scale bar: 10 μm in ***H*_3_**.

To examine the functional involvement of Arf4 and Arf5 in cortical development, we performed *in vivo* knock-down experiments using IUE. First, we designed shRNAs against Arf4 (shArf4#1, shArf4#2) and Arf5 (shArf5) by targeting divergent nucleotide sequences among Arf isoforms and validated their knock-down efficiency using immunoblotting (Fig. 3*A*,*B*). Primary cortical neurons prepared from E14 embryos were transfected with each shRNA plasmid by electroporation, maintained for 3 d, and subjected to immunoblotting with anti-Arf4 and Arf5 antibodies. The expression of each shRNA reduced endogenous protein expression of the respective Arf (Fig. 3*A*,*B*; Table 3; shArf4#1: 0.23 ± 0.002, *p *=* *0.0099; shArf4#2: 0.45 ± 0.291, *p *=* *0.0440; shArf5: 0.17 ± 0.09, *p *=* *0.0123) without compensatory upregulation of each other (Fig. 3*A*,*B*; Table 3; shArf4#1: Arf5, 1.06 ± 0.26, *p *=* *0.9674; shArf4#2: Arf5, 1.03 ± 0.43, *p *=* *0.9915; shArf5: Arf4, 1.88 ± 1.04, *p *= 0.2257). Further immunofluorescence analyses of E17 cerebral cortices electroporated with the shRNAs at E14 revealed that punctate immunofluorescence signals for Arf4 or Arf5 were significantly reduced in migrating neurons in the IZ (Fig. 3*D*,*H*), compared with that in control neurons (Fig. 3*C*,*F*; Arf4: Control, 1.0 ± 0.44, *n* = 24 cells; shArf4#1, 0.3 ± 0.17, *n* = 29 cells, *p *<* *0.0001; Arf5: Control, 1.0 ± 0.12, *n* = 8 cells; shArf5, 0.38 ± 0.11, *n* = 8 cells, *p *<* *0.0001). These results suggest that shRNAs against Arf4 and Arf5 specifically target the respective endogenous Arf proteins.

**Figure 4.**
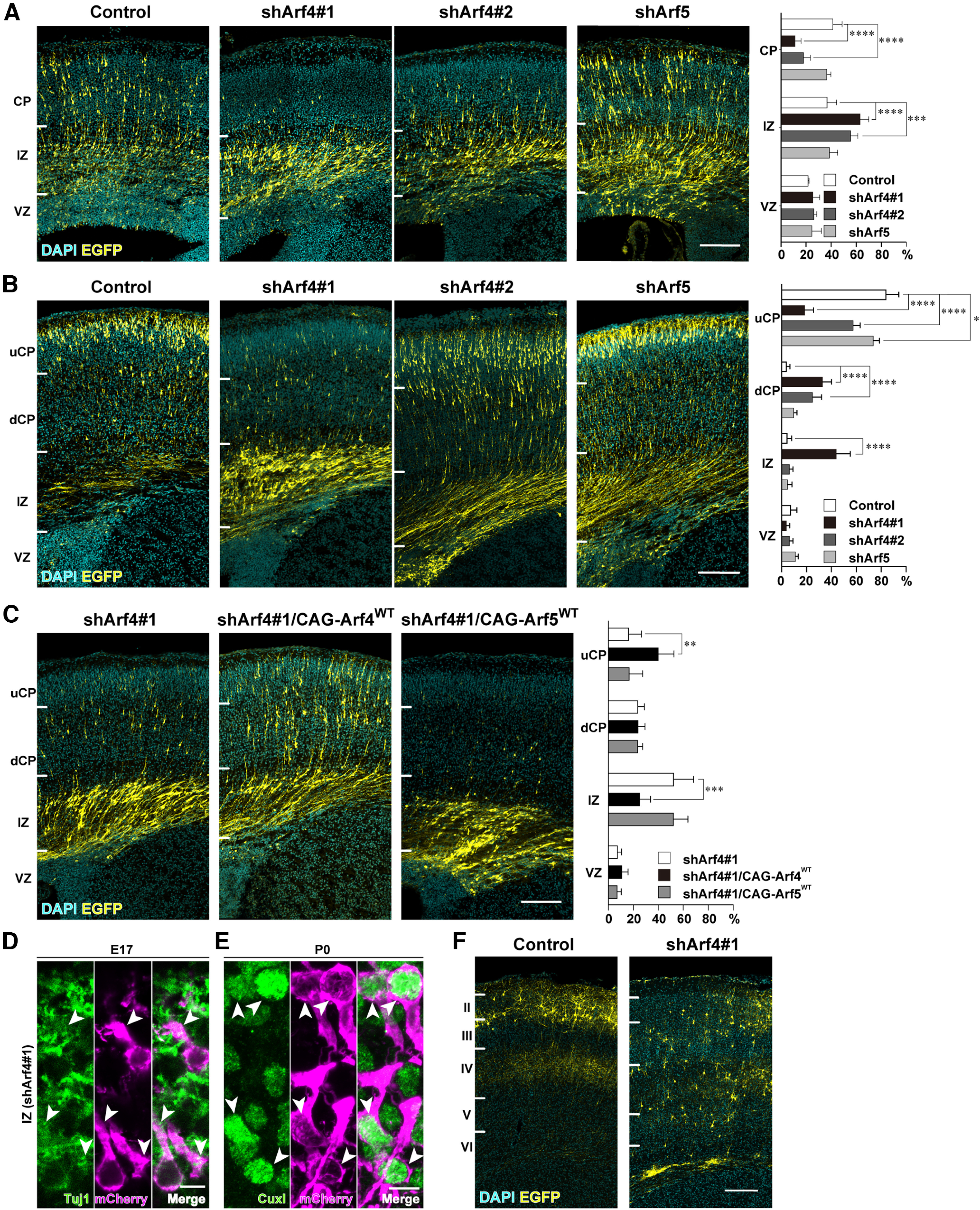
Knock-down of Arf4 disturbs cortical radial migration. ***A***, Representative micrographs of E17 cerebral cortices electroporated with the mU6pro plasmids for control shRNA, shArf4#1, shArf4#2, or shArf5, and pCAGGS-EGFP at E14 (Control, *n* = 4 embryos; shArf4#1, *n* = 5 embryos; shArf4#2, *n* = 5 embryos; shArf5, *n* = 4 embryos). The cortical wall was divided into three zones, i.e., cortical plate (CP), intermediate zone (IZ), and ventricular zone (VZ), by nuclear density. ***B***, Representative micrographs of P0 cerebral cortices electroporated with control, shArf4#1, shArf4#2, or shArf5 plasmids and pCAGGS-EGFP at E14 (Control, *n* = 5 embryos; shArf4#1, *n* = 4 embryos; shArf4#2, *n* = 4; shArf5, *n* = 5 embryos). Cortical wall was divided into four zones, i.e., upper cortical plate (uCP), deep cortical plate (dCP), IZ, and VZ by the combination of the immunoreactivity for Cux1 and nuclear density. ***C***, Representative micrographs of P0 cerebral cortices electroporated with shArf4#1 (*n* = 4 embryos), shArf4#1 and pCAGGS-shRNA-resistant wild-type Arf4 (CAG-Arf4^WT^; *n* = 4 embryos), or shArf4#1 and pCAGGS-wild-type Arf5 (CAG-Arf5^WT^; *n* = 4 embryos) plus pCAGGS-EGFP at E14. Note that migration defects caused by Arf4 knock-down could be partially rescued by coexpression of Arf4, but not Arf5. ***D***, ***E***, Representative immunofluorescence images showing the effect of Arf4 knock-down on the expression of Tuj1 (***D***) and Cux1 (***E***). Sections of the E17 (***D***) or P0 (***E***) cerebral cortices that had been electroporated with shArf4#1 and mCherry at E14 were subjected to double immunofluorescence with antibodies against Tuj1 (***D***) or Cux1 (***E***) and mCherry. Arrowheads indicate the expression of Tuj1 or Cux1 in shArf4#1-transfected cells visualized by mCherry immunofluorescence in the IZ. ***F***, Representative micrographs of P10 cerebral cortices electroporated with the control shRNA or shArf4#1 at E14. Note that control plasmid-transfected neurons were located in the Layer II or III, whereas shArf4#1-transfected neurons were scattered throughout the CP. Graphs in ***A*–*C*** show the quantification of the distribution of EGFP-positive cells in cortical zones. Data were presented as mean ± SD and statistically analyzed using two-way ANOVA followed by Tukey–Kramer’s test (*p < 0.05, **p < 0.05, ***p < 0.005, ****p < 0.0001). Scale bars: 200 μm in ***A*–*C***, and ***F***, 10 μm in ***D*** and ***E***.

Having established the efficiency of shRNAs against Arf4 and Arf5, we examined the role of Arf4 and Arf5 in radial migration during cortical development. Embryos electroporated with each shRNA and pCAGGS-EGFP at E14 were killed at 3 d (E17) or 5 d (P0) after IUE and the distribution of EGFP-positive cells in cortical zones was analyzed. The developing cerebral cortex was divided into three zones, VZ, IZ, and CP, at E17 based on nuclear density with DAPI and into four zones, VZ, IZ, dCP, and uCP, at P0 based on the combination of nuclear density and immunoreactivity for Cux1 as described in Materials and Methods. At E17, 41.5 ± 7.2% of the cells transfected with control shRNA were distributed in the CP (Fig. 4*A*; Table 3; IZ, 36.8 ± 7.5%, VZ, 21.7 ± 0.6%, *n* = 4). At P0, 84.0 ± 10.4% of the control cells were distributed in the uCP (Fig. 4*B*; dCP, 4.1 ± 2.6%, IZ, 4.6 ± 3.5%, VZ, 7.3 ± 5.0%, *n* = 5). In contrast, Arf4 knock-down using shArf4#1 significantly inhibited the entry of migrating neurons into the CP with marked accumulation of transfected cells in the IZ at E17 (Fig. 4*A*; Table 3; shArf4#1: CP, 11.3 ± 4.7%, *p *<* *0.0001, IZ, 63.3 ± 6.8%, *p *<* *0.0001, VZ, 25.4 ± 5.4%, *p *=* *0.7489, *n* = 5). At P0, most Arf4#1-transfected cells still remained in the dCP and IZ (Fig. 4*B*; Table 3; shArf4#1: uCP, 18.8 ± 7.0%, *p *<* *0.0001, dCP, 33.1 ± 7.1%, *p *<* *0.0001, IZ, 44.1 ± 11.0%, *p *<* *0.0001, VZ, 4.1 ± 2.3%, *p *=* *0.8241, *n* = 4). An independent shRNA against Arf4 (shArf4#2), which showed a milder knock-down efficiency (Fig. 3*A*), had similar inhibitory effects on the distribution of transfected cells in cortical zones at E17 and P0, except for the absence of significant accumulation of transfected cells in the IZ at P0 (Fig. 4*A*,*B*; Table 3; E17: CP, 18.1 ± 5.4%, *p *<* *0.0001, IZ, 55.6 ± 5.7%, *p *<* *0.0001, VZ, 26.4 ± 2.1%, *p *=* *0.5971, *n* = 5; P0: uCP, 57.6 ± 5.5%, *p *<* *0.0001, dCP, 25.0 ± 7.2%, *p *<* *0.0001, IZ, 6.4 ± 2.7%, *p *=* *0.9624, VZ, 10.9 ± 1.7%, *p *=* *0.9948, *n* = 4). This phenotypic discrepancy in the IZ between the two shRNAs for Arf4 may be explained by the difference in their knock-down efficiencies. In contrast, knock-down of Arf5 did not have apparent effect on the distribution of transfected cells in cortical zones at E17, compared with that of control cells (Fig. 4*A*,*B*; Table 3; CP, 36.5 ± 3.2%, *p *=* *0.5882, IZ, 38.7 ± 6.5%, *p *=* *0.9654, VZ, 24.5 ± 7.5%, *p *=* *0.8575, *n* = 4). Interestingly, at P0, there was a mild, but significant, reduction in the proportion of shArf5-transfected cells in the uCP (Fig. 4*B*; Table 3; uCP, 72.8 ± 4.8%, *p *=* *0.0300, dCP, 10.9 ± 1.3%, *p *=* *0.3648, IZ, 4.8 ± 4.0%, *p *=* *0.9998, VZ, 11.5 ± 2.4%, *p *=* *0.6709, *n* = 5), suggesting that Arf5 may play a role in terminal translocation, which will be a subject of the future study.

To exclude the off-target effect of shArf4, we performed the rescue experiment and showed that the migration defect caused by Arf4-knock-down was partially rescued by the co-transfection of shRNA-resistant wild-type Arf4 (Fig. 4*C*; Table 3; shArf4#1 plus Arf4: uCP, 40.0 ± 12.7%, *p *=* *0.0017, dCP, 24.0 ± 5.5%, *p *=* *0.9998, IZ, 25.1 ± 8.6%, *p *=* *0.0003, VZ, 10.8 ± 5.0%, *p* = 0.8499, *n* = 4; compared with shArf4#1; shArf4#1: uCP, 16.3 ± 10.0%, dCP, 23.9 ± 5.0%, IZ, 52.4 ± 16.0%, VZ, 7.4 ± 3.1%, *n* = 4), but not wild-type Arf5 (Fig. 4*C*; Table 3; shArf4#1 plus Arf5: uCP, 16.8 ± 10.6%, *p *=* *0.9991, dCP, 23.6 ± 3.8%, *p *=* *0.9986, IZ, 52.3 ± 11.4%, *p *=* *0.0003; VZ, 7.2 ± 3.1%, *p *=* *0.8499, *n* = 4), suggesting that Arf4 plays a distinct role from Arf5 in cortical neuronal migration.

Furthermore, immunofluorescence staining with Tuj1 (Class III β-tubulin) and Cux1, differentiation markers for neurons and cortical Layers II–IV excitatory neurons, respectively, revealed that Arf4-knock-down cells in the IZ were immunoreactive for Tuj1 at E17 and Cux1 at P0 to the same extent as surrounding neurons and cortical Layer II/III neurons, respectively (Fig. 4*D*,*E*, arrows), suggesting that Arf4 knock-down did not affect neuronal differentiation.

Finally, we examined the distribution of Arf4 knock-down cells in the P10 cerebral cortex that had been electroporated with shArf4#1 and pCAGGS-EGFP at E14. Arf4-knock-down neurons were still observed in the lower cortical layer at P10 compared with the control, suggesting that Arf4 knock-down led to a permanent defect in radial migration (Fig. 4*F*).

### Knock-down of Arf4 or Arf5 does not affect cell proliferation

**Figure 5.**
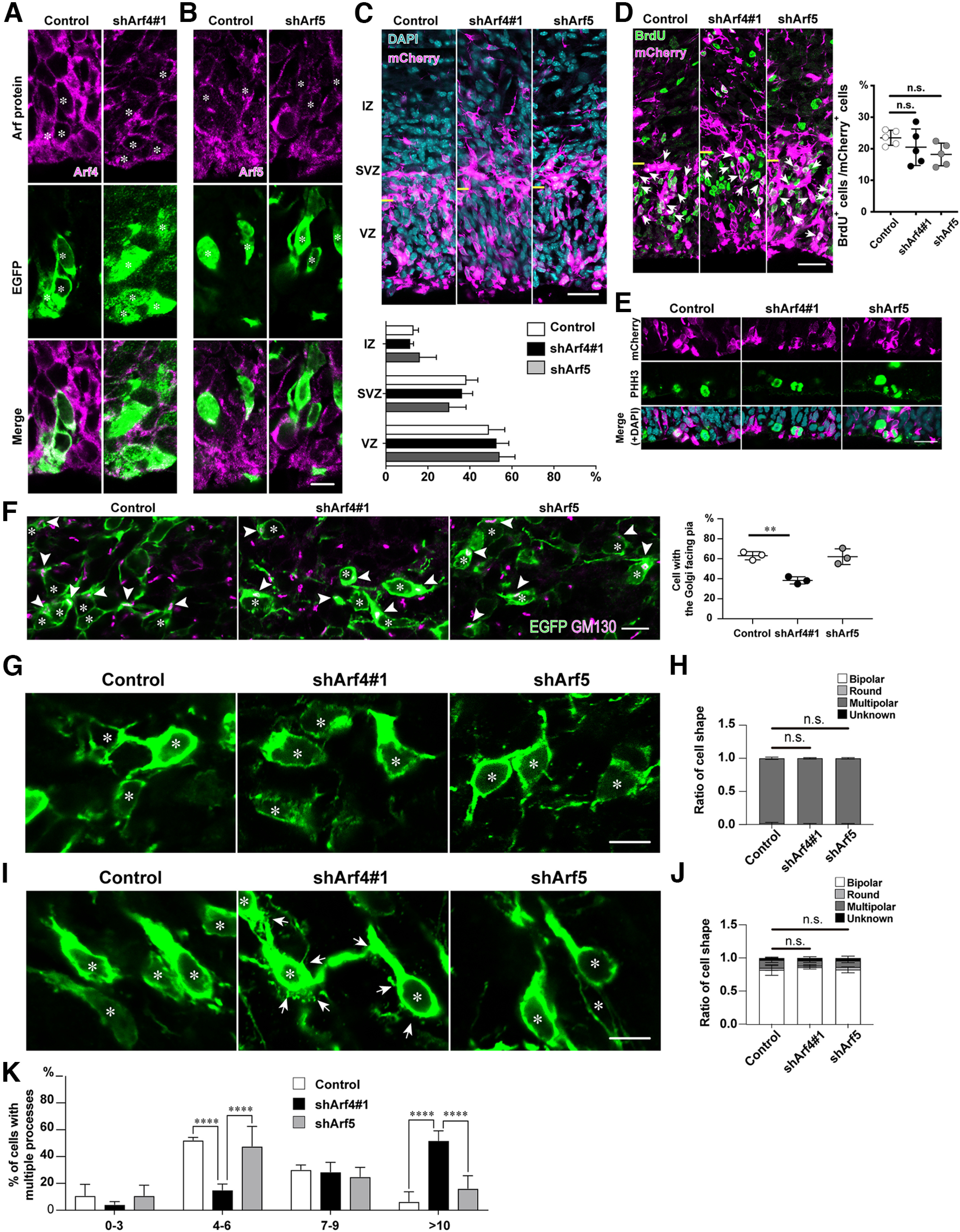
Knock-down of Arf4 disturbs the Golgi orientation, but not multipolar-to-bipolar morphologic transition in the IZ. ***A***, ***B***, Representative immunofluorescence images of the ventricular zone at E15 at 1 d after electroporation with indicated shRNA and EGFP. Note that ventricular cells transfected with shArf4#1 (***A***) or shArf5 (***B***) exhibited reduced endogenous expression of the respective Arf. Asterisks in ***A*** and ***B*** indicate the nuclei of transfected cells. ***C***, Representative micrographs of E15 cerebral cortices electroporated with control, shArf4, or shArf5 plasmids plus pCAGGS-mCherry. The VZ, SVZ, and IZ were divided by the immunoreactivity for Sox2 and nuclear density. Yellow bars indicate border between the VZ and SVZ. The graph shows the percentage of transfected cells in each zone. ***D***, Representative micrographs showing the effect of knock-down of Arf4 or Arf5 on BrdU incorporation in the VZ at E15. Embryos were electroporated with indicated shRNA plasmids plus pCAGGS-mCherry at E14, killed at E15 after BrdU administration, and immunostained with antibodies against mCherry (magenta) and BrdU (green). Arrows indicate the BrdU incorporation in transfected cells. Yellow bars indicate border between the VZ and SVZ. The graph shows the percentage of BrdU-incorporated cells in total transfected cells. ***E***, Representative micrographs showing the effect of knock-down of Arf4 or Arf5 on phospho-histone H3 (PHH3)-positive cells in the VZ at E15. Note no differences in the proportion of PHH3-positive mitotic cells among the control, shArf4#1-transfected, or shArf5-transfected cells in the VZ. ***F***, The orientation of the Golgi apparatus in migrating neurons in the lower IZ. Embryos were electroporated with indicated plasmids and pCAGGS-EGFP at E14, killed at E16, and immunostained with antibodies against GM130 (magenta) and EGFP (green). Arrowheads and asterisks indicate the Golgi apparatus and nuclei, respectively, in transfected cells. The graph shows the percentage of cells with the Golgi facing the CP in total EGFP-positive transfected cells in the IZ. ***G***, ***I***, High magnification of EGFP-positive multipolar migrating neurons in the lower IZ at E16 (***G***) and upper IZ at E17 (***I***). Asterisks in ***G*** and ***I*** indicate the nuclei of transfected cells. Arrows in ***I*** indicate filopodia-like fine processes extending from the cell body and leading process of shArf4#1-transfected cells. ***H***, ***J***, Graphs show that the proportion of the cell morphology of EGFP-positive cells in the lower IZ at E16 (***H***) and in the upper IZ (***J***). Note no significant effect of either Arf4 or Arf5 knock-down on multipolar-to-bipolar morphologic transition. ***K***, Graph showing the proportion of the number of processes extending from the cell bodies in bipolar cells in the upper IZ at E17 transfected with control, shArf4#1, or shArf5. Data were presented as mean ± SD and statistically analyzed using one-way (***D***, ***F***) or two-way (***C***, ***H***, ***J***, ***K***) ANOVA followed by Tukey–Kramer’s test (**p <* *0.005, ****p <* *0.0001, n.s., not significant). Scale bars: 30 μm in ***C*** and ***D***, 20 μm in ***E***, and 10 μm in ***B***, ***F***, ***G***, and ***I***.

Disturbed neuronal positioning in the developing cerebral cortex with accumulation of shArf4-transfected cells in the IZ could be caused by defects in migration as well as other cellular processes, including cell cycle progression and exit from the VZ. First, we examined whether knock-down effect started to take place in the E15 ventricular zone at 1 d after IUE. Immunohistological analyses of the ventricular zone revealed that immunofluorescence intensities of Arf4 and Arf5 were decreased to 0.53 ± 0.17 (*n* = 37 cells, *p *<* *0.0001; Control: 1.0 ± 0.26, *n* = 33 cells) and 0.24 ± 0.16 (*n* = 21 cells, *p *<* *0.0001; Control: 1.0 ± 0.51, *n* = 21 cells) in shArf4#1-transfected and shArf5-transfected cells, respectively, compared with that in the control cells (Fig. 5*A*,*B*). Next, to examine the effect of Arf4 knock-down on the cell cycle progression in the VZ and the exit from VZ, pregnant mice were intraperitoneally administrated with BrdU at E15, 1 d after IUE with shArf4#1 or shArf5 and mCherry. The embryos were killed 15 min after the final BrdU injection and subjected to immunostaining with EGFP and BrdU. At E15, most of the control and Arf4/5-knock-down cells similarly distributed in the VZ and SVZ (Fig. 5*C*; Table 3; Control: IZ, 13.0 ± 2.5%, SVZ, 38.2 ± 5.6%, VZ, 48.8 ± 7.7%, *n* = 5; shArf4#1: IZ, 11.4 ± 1.7%, *p *=* *0.5028, SVZ, 36.1 ± 5.2%, *p *=* *0.8222, VZ, 52.5 ± 6.0%, *p *=* *0.6921, *n* = 5; shArf5: IZ, 16.0 ± 8.0%, *p *=* *0.7215, SVZ, 30.0 ± 8.2%, *p *= 0.2274, VZ, 54.0 ± 7.5%, *p *=* *0.5604, *n* = 5), suggesting that knock-down of Arf4 or Arf5 did not affect the exit of progenitor cells from the VZ to the SVZ. Quantification of the percentage of BrdU-incorporated cells in transfected cells in the VZ was comparable between the control and Arf4 or Arf5 knock-down (Fig. 5*D*; Table 3; Control: 23.5 ± 2.4%, *n* = 5; shArf4#1: 20.5 ± 5.8%, *n* = 5, *p *=* *0.5074; shArf5: 18.2 ± 3.6%, *n* = 5, *p *=* *0.1526). Furthermore, there were no significant differences in the percentages of ventricular epithelial cells labeled by an antibody against phospho-histone H3, a mitosis-specific marker, in transfected cells between the control and knock-down (Fig. 5*E*; Control: 13.7 ± 4.2%, *n* = 5; shArf4#1: 14.7 ± 2.7%, *n* = 4, *p *= 0.9136; shArf5: 10.9 ± 4.1%, *n* = 3, *p *=* *0.586). These results suggest that stalling of Arf4-knock-down cells in the IZ was unlikely to be caused by disturbances in cell cycle progression of cortical progenitor cells or the exit of ventricular progenitor cells from the VZ.

### Arf4, but not Arf5, regulates multipolar migration in the IZ

During multipolar migration in the IZ, migrating neurons establish polarity and undergo a dynamic morphologic transition from a multipolar to bipolar shape (Barnes and Polleux, 2009; Cooper, 2014). We first examined the orientation of Golgi apparatus by immunostaining of migrating neurons in the lower IZ using an anti-GM130 antibody. In the lower IZ at E16, most multipolar cells electroporated with control shRNA and EGFP at E14 had a supranuclear Golgi apparatus oriented toward the CP (Fig. 5*F*; 63.1 ± 4.1%). In contrast, multipolar cells transfected with shArf4#1, but not shArf5, exhibited the Golgi positioned juxtanuclearly, but with varying orientations (Fig. 5*F*; shArf4#1: 38.4 ± 3.6%, *p *=* *0.0037, *n* = 3; shArf5: 62.1 ± 7.8%, *p *=* *0.9768, *n* = 3).

We also examined whether knock-down of Arf4 or Arf5 regulates the transition from multipolar to bipolar morphology in neurons migrating in the IZ by classifying transfected cells as multipolar, round, and bipolar shapes. At E16, most of control migrating cells in the lower IZ exhibited multipolar morphology with multiple processes extending from the cell bodies in various directions, and transformed to bipolar morphology with a leading processes extending toward the pial direction at the upper IZ at E17 (Fig. 5*H*,*J*) Quantification of the cell morphology revealed that there were no significant differences in the proportion of cell shapes among the control, shArf4#1-transfected, and shArf5-transfected migrating cells in the IZ at E16 and E17 (Fig. 5*H*,*J*; Table 3). However, it should be noted that Arf4-knock-down bipolar cells in the upper IZ at E17 possessed numerous filopodia-like, fine, short processes extending from their cell bodies and leading processes (Fig. 5*I*, arrows). Quantification revealed that Arf4#1-transfected cells possessed more short processes extending from the cell body than the control or shArf5-transfected cells (Fig. 5*K*; Table 3). These findings suggest that Arf4 regulates the Golgi polarization and cell morphology, although it is not involved in multipolar-to-bipolar morphologic transition.

**Figure 6.**
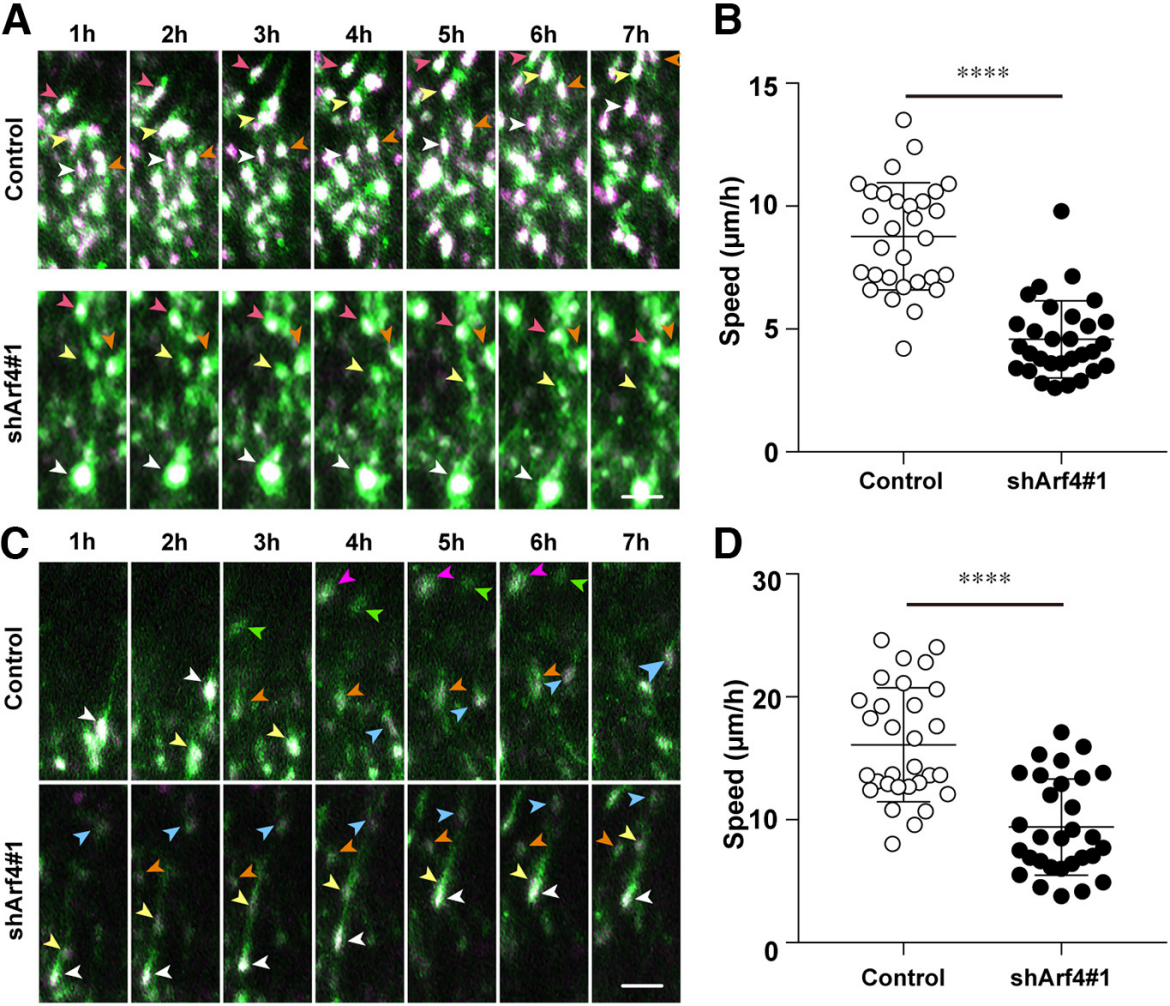
Knock-down of Arf4 reduces migration speed in the IZ and CP. ***A***, ***C***, Representative time-lapse images of transfected neurons migrating in the lower IZ (***A***) and CP (***C***) at E17. Embryos were electroporated with the indicated shRNA plus pCAGGS-FloxP-EGFP, pCAGGS-FloxP-EGFP-F, pCAGGS-FloxP-mCherry-NLS, and pCAGGS-Cre recombinase at E14, and brains were subjected to slice culture and time-lapse observation at E17. Arrowheads indicate the nuclei of transfected neurons. ***B***, ***D***, Quantification of the migration speed of neurons transfected with indicated shRNAs in the lower IZ (***B***) and CP (***D***). Note the reduction of the migration speed in shArf4-transfected cells in the lower IZ and CP, compared with that in control cells. Data were presented as mean ± SD and statistically analyzed using Mann–Whitney *U* test (****p <* *0.0001). Scale bars: 30 μm in ***A*** and ***C***.

Furthermore, time-lapse imaging of an organotypic brain slice culture from E17 embryos electroporated at E14 revealed that Arf4 knock-down significantly reduced the speed of multipolar migration in the lower IZ at E17, compared with that of the control (Fig. 6*A*,*B*; Table 3, Control: 8.8 ± 2.2 μm/h, *n* = 30 cells, 3 embryos from 2 pregnant mice; shArf4#1: 4.6 ± 1.6 μm/h, *n* = 30 cells, 3 embryos from 2 pregnant mice, *p *<* *0.0001). These results suggest that Arf4 also regulates cell motility during multipolar migration in the IZ.

### Arf4, but not Arf5, also regulates locomotion in the CP

Because Arf4 knock-down reduced the proportion of cells that reached the upper CP at P0 (Fig. 4*B*), we examined the effect of Arf4 knock-down on radial migration behaviors in the CP using time-lapse imaging of an organotypic brain slice culture. Arf4 knock-down significantly reduced the speed of locomotion toward the pia, compared with that in the control (Fig. 6*C*,*D*; Table 3; Control: 16.1 ± 4.6 μm/h, *n* = 30 cells, 3 embryos from 2 pregnant mice; shArf4#1: 9.4 ± 3.9 μm/h, *n* = 30 cells, 3 embryos from 2 pregnant mice, *p *<* *0.0001), suggesting that Arf4 regulates cell motility during locomotion in the CP as well as multipolar migration in the IZ.

### Knock-down of Arf4 or Arf5 affects the morphology of the Golgi and endosomes in migrating neurons

**Figure 7.**
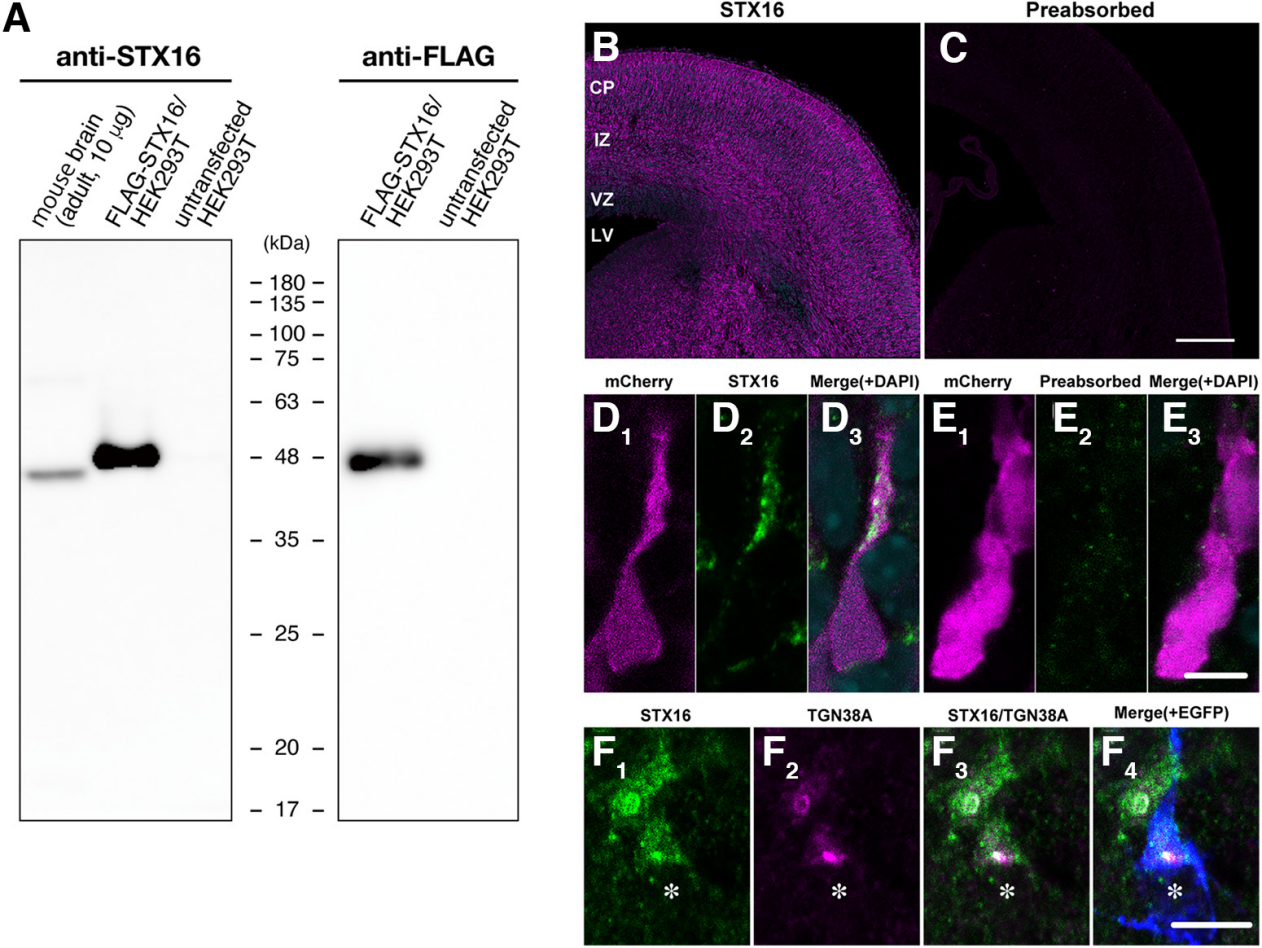
Characterization of an anti-STX16 antibody. ***A***, Characterization of an anti-STX16 antibody. The lysates of the mouse brain and HEK293T cells transfected or untransfected with pCAGGS-FLAG-STX16 were subjected to immunoblotting with antibodies against anti-STX16 (left) or anti-FLAG (right). ***B*–*E***, Representative micrographs of STX16 immunoreactivity in E17 cerebral cortices (***B***, ***C***) and migrating neurons at a high magnification (***D***, ***E***). Sections of E17 cerebral cortices that had been transfected pCAGGS-mCherry at E14 were immunostained with antibody against mCherry (***D*_1_**, ***E*_1_**) and STX16 (***B***, ***D*_2_**) or antibody preabsorbed with STX16 (***C***, ***E*_2_**). ***F***, Subcellular localization of STX16 in migrating neurons. Sections of E17 cerebral cortices that had been electroporated with pCAGGS-mCherry were immunostained with antibodies against STX16 (***F*_1_**, green), TGN38A (***F*_2_**, magenta), and mCherry (***F*_4_**, blue). Note the partial colocalization of STX16 with TGN38A at the juxtanuclear region. An asterisk indicates the nucleus of a transfected migrating neuron in the IZ. Scale bars; 200 μm in ***C***, 10 μm in ***E*** and ***F***.

We examined whether knock-down of Arf4 or Arf5 affected the morphology of organelles related to secretory and endocytic pathways, including the Golgi, TGN, and endosomes, in migrating neurons by immunostaining with antibodies against GM130, STX16, VAMP4, STX12, Rab11, and EEA1. The specificity of an anti-STX16 antibody was confirmed by immunoblotting in which the antibody detected an immunoreactive band of 45–48 kDa in the lysates of mouse brains and HEK293T cells transfected with FLAG epitope-tagged STX16 (Fig. 7*A*). The immunoreactivity of STX16 was detected in the entire E17 cerebral cortex (Fig. 7*B*) and migrating neurons visualized by mCherry (Fig. 7*D*), which was completely attenuated by preabsorption of the antibody with STX16 (Fig. 7*C*,*E*). Furthermore, the antibody labeled punctate structures partially overlapped and/or closely associated with TGN38A in migrating neurons visualized by EGFP in the upper IZ (Fig. 7*F*). These findings suggested the specificity of the newly generated anti-STX16 antibody.

**Figure 8.**
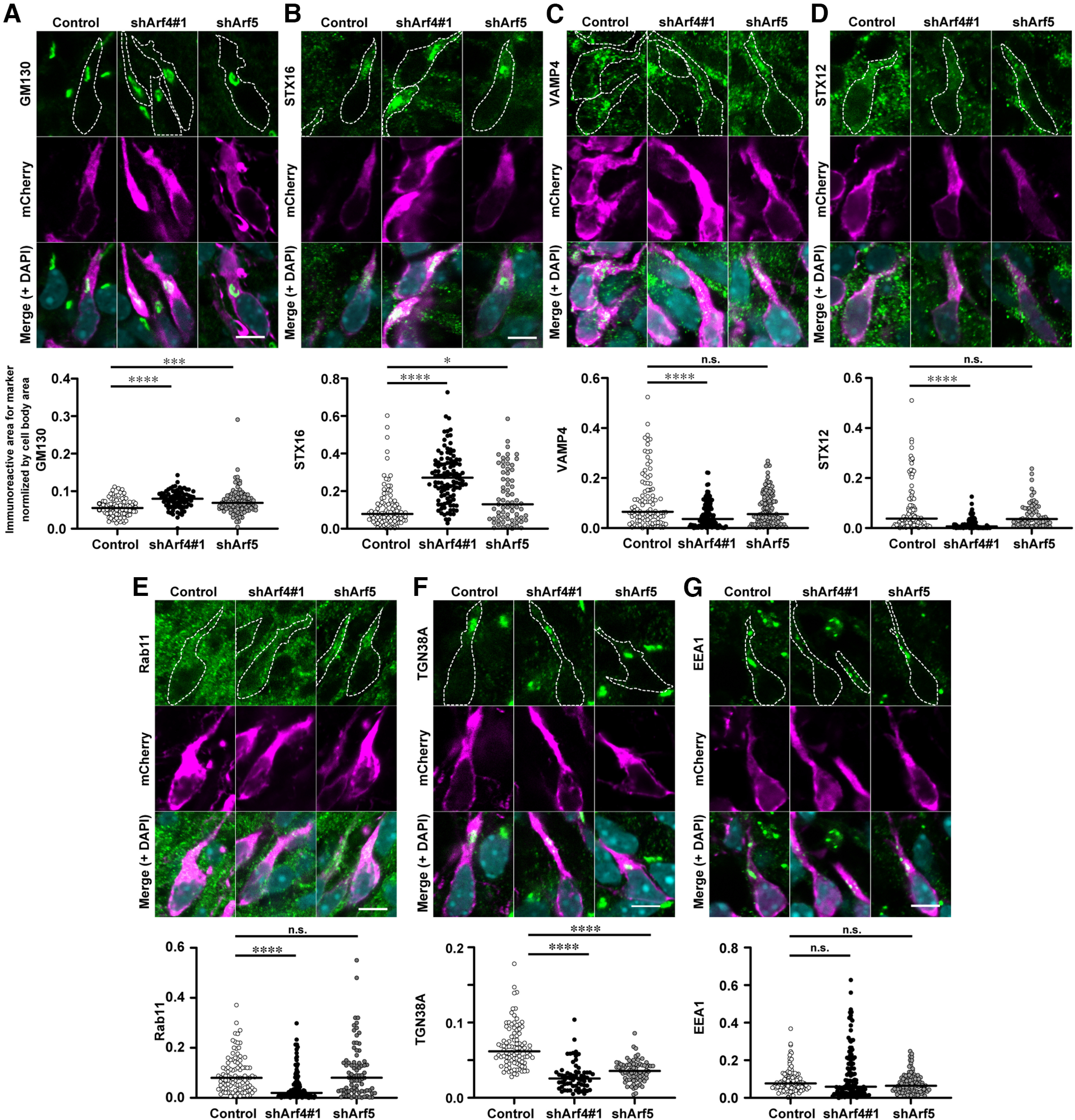
Knock-down of Arf4 or Arf5 affects the morphology and distribution of the Golgi and endosomes in migrating neurons. ***A*–*G***, Representative micrographs showing the morphology of organelles and endosomes in transfected neurons in the IZ at E17. Cerebral cortices electroporated with indicated shRNAs and pCAGGS-mCherry at E14 were fixed at E17 and subjected to double immunofluorescence staining with antibodies against mCherry (magenta) and marker proteins (green), including GM130 (***A***), STX16 (***B***), VAMP4 (***C***), STX12 (***D***), Rab11 (***E***), TGN38A (***F***), or EEA1 (***G***). The lower graphs show the quantification of the immunoreactive areas for markers normalized by the cell body area. Note that Arf4 knock-down specifically reduced the immunoreactive areas for VAMP4, STX12, and Rab11, whereas either Arf4 or Arf5 knock-down increased the immunoreactive areas for TGN38A and STX16. Broken lines indicate the contour of transfected cells visualized by mCherry immunofluorescence. Data were presented as mean ± SEM and statistically analyzed using Kruskal–Wallis test followed by Dunn’s multiple comparison test (*p <* *0.05, ***p <* *0.005, ****p <* *0.0001, n.s., not significant). Scale bars: 10 μm in ***A*–*G***.

We then performed immunofluorescence analyses to examine the effect of Arf4 or Arf5 knock-down on the distribution of organelle markers in migrating neurons by quantifying the immunoreactive areas for markers normalized by cell body areas. Knock-down of either Arf4 or Arf5 resulted in enlargement of the Golgi apparatus visualized by GM130 without apparent morphologic changes, such as fragmentation or dispersion (Fig. 8*A*; Table 3; Control: 0.06 ± 0.002, *n* = 90 cells; shArf4#1: 0.08 ± 0.003, *n* = 77 cells, *p < *0.0001; shArf5: 0.07 ± 0.003, *n* = 102 cells, *p *=* *0.0006). Knock-down of Arf4 or Arf5 also increased the immunoreactive area for STX16, a member of the soluble N-ethylmalemide-sensitive factor attachment protein receptor (SNARE) family localized primarily at the TGN, with Arf4 knock-down more potently (Fig. 8*B*; Table 3; Control: 0.11 ± 0.011, *n* = 97 cells; shArf4#1: 0.27 ± 0.013, *n* = 109 cells, *p *<* *0.0001; shArf5: 0.17 ± 0.017, *n* = 69 cells, *p *=* *0.0068). Notably, knock-down of Arf4, but not Arf5, significantly decreased the immunoreactive area for VAMP4, a partner SNARE protein for sytaxin16 localized mainly on transport vesicles (Fig. 8*C*; Table 3; Control: 0.011 ± 0.1, *n* = 99 cells; shArf4#1: 0.05 ± 0.005, *n* = 114 cells, *p < *0.0001; shArf5: 0.08 ± 0.006, *n* = 120 cells, *p *=* *0.4254), STX12, a SNARE protein localized mainly on recycling endosomes (Fig. 8*D*; Table 3; Control: 0.08 ± 0.011, *n* = 93 cells; shArf4#1: 0.02 ± 0.002, *n* = 94 cells, *p *<* *0.0001; shArf5: 0.06 ± 0.006, *n* = 71 cells, *p *>* *0.9999), and Rab11, a small GTPase localized on recycling endosomes (Fig. 8*E*; Table 3; Control: 0.09 ± 0.007, *n* = 104 cells; shArf4#1: 0.05 ± 0.005, *n* = 121 cells, *p < *0.0001; shArf5: 0.11 ± 0.012, *n* = 80 cells, *p *= 0.8966). In addition, knock-down of Arf4 or Arf5 significantly decreased the immunoreactive perinuclear area for TGN38A (Fig. 8*F*; Table 3; Control: 0.07 ± 0.003, *n* = 98 cells; shArf4#1: 0.03 ± 0.002, *n* = 64 cells, *p *<* *0.0001; shArf5: 0.04 ± 0.002, *n* = 75 cells, *p *<* *0.0001). In contrast, knock-down of either Arf4 or Arf5 did not affect the immunoreactive area for EEA1 (Fig. 8*G*; Table 3; Control: 0.09 ± 0.01, *n* = 103 cells; shArf4#1, 0.12 ± 0.01, *n* = 126 cells, *p *=* *0.5118; shArf5: 0.08 ± 0.01, *n* = 105 cells, *p *=* *0.1680). These results suggest that Arf4 plays overlapping but distinct roles from Arf5 in the morphology and/or distribution of endosomal compartments related to the retrograde transport to the TGN and recycling to the plasma membrane.

### Arf4 regulates radial migration through N-cadherin trafficking

**Figure 9.**
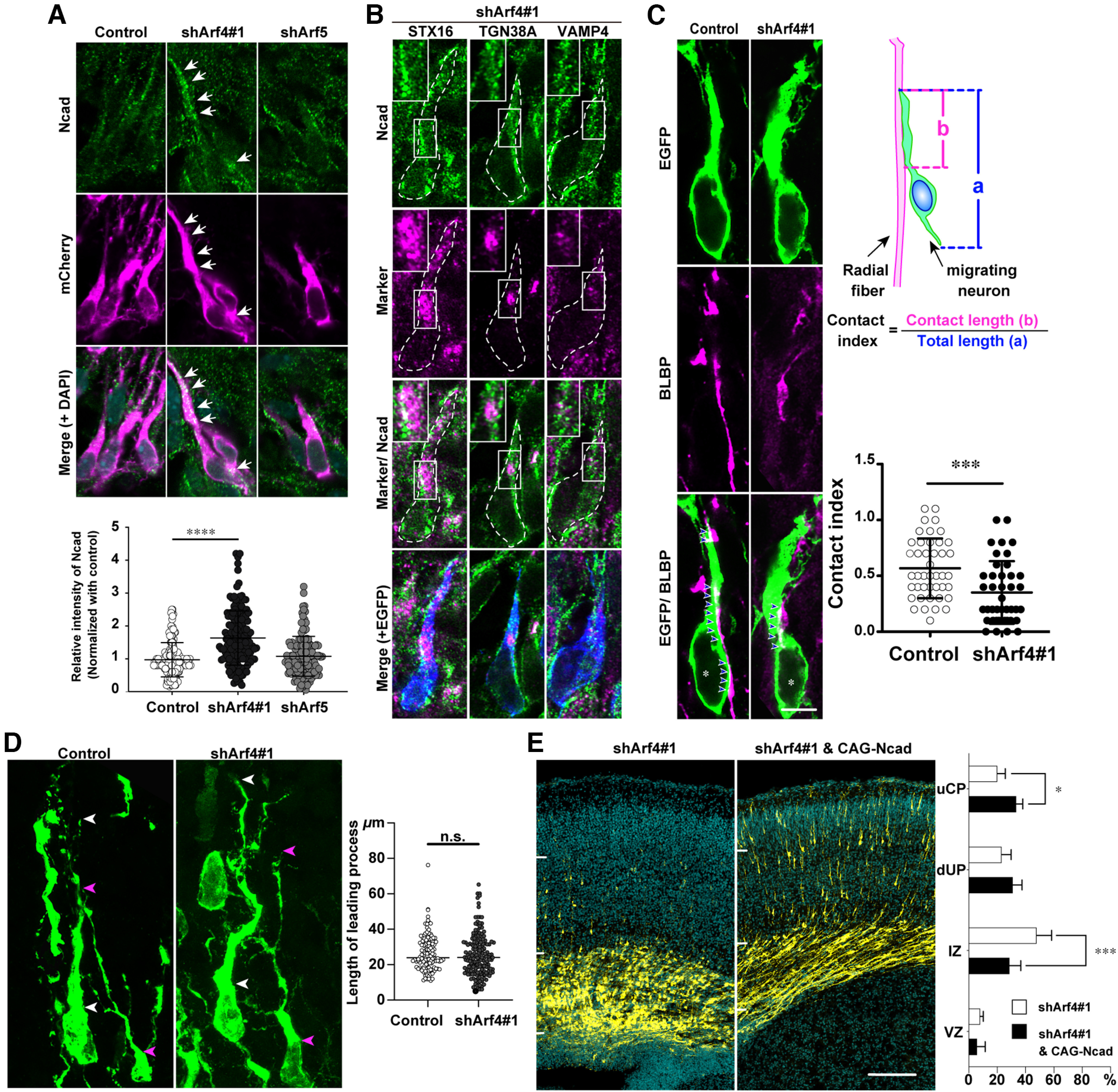
Arf4 regulates neuronal migration through N-cadherin trafficking. ***A***, Representative micrographs showing the effect of knock-down of Arf4 or Arf5 on the subcellular localization of N-cadherin in migrating neurons. Brains were electroporated with shArf4 or shArf5 plus mCherry, killed at E17, and subjected to immunostaining with antibodies against N-cadherin (Ncad; green) and mCherry (magenta). Arrows indicate intracellular N-cadherin in shArf4#1 transfected cells. The lower graph shows the relative immunofluorescence intensity of intracellular N-cadherin to that of the control. Note the cytoplasmic accumulation of N-cadherin in migrating neurons transfected with shArf4#1, but not shArf5, in the IZ at E17. ***B***, Representative images of triple immunofluorescence staining of Arf4-knock-down neurons in the IZ with antibodies against N-cadherin (green), STX16 (magenta), TGN38A (magenta), or VAMP4 (magenta), and EGFP (blue). Boxed areas show the colocalization of N-cadherin with STX16, TGN38A, or VAMP4 in transfected neurons at a high magnification. Broken lines indicate the contour of transfected cells visualized by EGFP immunofluorescence. ***C***, Representative images of bipolar neurons migrating along BLBP-immunoreactive radial fibers (magenta) in the upper IZ. Arrowheads indicate the contact of EGFP-positive transfected neurons with BLBP-positive radial fibers. The cartoon illustrates the contact index, which is calculated by dividing the length of the contact between an EGFP-positive migrating neuron and BLBP-immunoreactive radial glial fibers by the total length of the cell body and leading process of the transfected neuron observed on a single image. A graph shows the quantification of the contact index. Note that Arf4 knock-down significantly reduced the cell-cell adhesion between migrating neurons and radial glial fibers. ***D***, Representative images of the morphology of bipolar neurons with a leading process in the upper IZ at E17. Arrowheads indicate the distal tip and proximal base of leading processes of transfected bipolar cells. A graph shows the effect of Arf4 knock-down on the length of leading processes. ***E***, Representative micrographs showing the effect of coexpression of N-cadherin with shArf4 on the migration defect caused by Arf4 knock-down. Embryos were subjected to *in utero* electroporation with shArf4#1 or shArf4#1 plus pCAGGS-N-cadherin (CAG-Ncad) at E14 and killed at P0. The graph shows the quantification of the distribution of EGFP-positive cells in cortical zones. Data were presented as mean ± SD and statistically analyzed using Kruskal–Wallis test followed by Dunn’s multiple comparison test (***A***, ****p <* *0.0001), Mann–Whitney *U* test (***C***, ***D***, ***p <* *0.005, n.s. not significant), or two-way ANOVA followed by Bonferroni’s multiple comparison test (***E***, *p <* *0.05, ***p <* *0.001). Scale bars: 10 μm in ***A–C***, 200 μm in ***D***.

N-cadherin trafficking is required for various steps of radial migration during cortical layer formation (Kawauchi et al., 2010; Shikanai et al., 2011; Martinez-Garay et al., 2016; Martinez-Garay, 2020). We have previously shown that Arf6 regulates multipolar migration through N-cadherin recycling (Hara et al., 2016). Therefore, we examined the effect of Arf4 knock-down on N-cadherin subcellular localization in migrating neurons by immunofluorescence staining of E17 embryos electroporated with shRNA and mCherry at E14 (Fig. 9*A*). In control neurons, N-cadherin-immunoreactive dots were distributed primarily along the surface of their cell bodies and leading processes. In contrast, knock-down of Arf4, but not Arf5, induced cytoplasmic accumulation of N-cadherin-positive dots in migrating neurons in the upper IZ. Quantitative analysis confirmed that knock-down of Arf4, not but Arf5, significantly increased the immunofluorescence intensity for N-cadherin inside the cell by 60%, compared with that of control shRNA (Fig. 9*A*; Table 3; Control: 1.0 ± 0.6, *n* = 110 cells; shArf4, 1.6 ± 0.9, *n* = 119 cells, *p < *0.0001; shArf5, 1.1 ± 0.6, *n* = 102 cells, *p *=* *0.4333). Furthermore, triple immunofluorescence staining and analyses of colocalization coefficient demonstrated that N-cadherin was present juxtanuclearly partially on STX16-positive, TGN38A-positive, and VAMP4-positive structures in migrating neurons (Fig. 9*B*; Table 3; STX16: 0.70 ± 0.27, *n* = 33 cells; TGN38: 0.95 ± 0.09, *n* = 45 cells; VAMP4: 0.62 ± 0.21, *n* = 33 cells), suggesting that intracellular N-cadherin were accumulated around the TGN in Arf4- knock-down cells.

Since N-cadherin mediates cell-cell adhesion between radially migrating neurons and radial glial fibers (Kawauchi et al., 2010; Martinez-Garay et al., 2016), we examined the effect of Arf4 knock-down on their interactions by calculating the contact index, which was defined by the ratio of contact length of EGFP-positive transfected migrating neurons with BLBP-immunoreactive radial glial fibers to the total length of their cell bodies and leading processes as shown in Figure 9*C*. Arf4 knock-down significantly decreased the contact index by 33%, compared with that of control shRNA (Fig. 9*C*; Table 3; Control: 0.6 ± 0.3, *n* = 43 cells; shArf4#1: 0.4 ± 0.3, *n* = 41 cells, *p *=* *0.0004). Furthermore, we examined the effect of Arf4 knock-down on the length of leading processes. However, there were no significant differences in leading process length between control and shArf4#1-transfected neurons (Fig. 9*D*; Table 3; Control: 26.7 ± 9.8 μm, *n* = 126 cells; shArf4#1: 25.5 ± 11.9 μm, *n* = 178 cells, *p = *0.1696). These results suggest that Arf4 regulates N-cadherin-mediated interaction with radial glial fibers in migrating neurons.

Finally, to examine whether supplementation of exogenous N-cadherin can rescue the migration defect caused by Arf4 knock-down, N-cadherin was co-electroporated with shArf4#1 and EGFP into embryos at E14. Coexpression of N-cadherin with shArf4#1 significantly increased the proportion of cells in the upper CP with a concomitant decrease in the IZ at E17, compared with that observed with shArf4 transfection alone (Fig. 9*E*; Table 3; shArf4#1: uCP, 20.3 ± 5.4%, dCP, 23.4 ± 6.5%, IZ, 48.0 ± 10.4%, VZ, 8.2 ± 2.0%, *n* = 4; shArf4#1 plus N-cadherin: uCP, 33.7 ± 4.3%, *p *=* *0.0165, dCP, 31.2 ± 6.3%, *p *=* *0.3022, IZ, 29.0 ± 7.7%, *p *=* *0.0005, VZ, 5.1 ± 5.4%, *p *>* *0.9999, *n* = 5). These results suggest that Arf4 partially regulates neuronal migration through N-cadherin trafficking to the cell surface.

## Discussion

In this study, we investigated the impact of Class II Arfs on cortical radial migration using IUE. Our results demonstrated that knock-down of Arf4, but not Arf5, led to an accumulation of transfected neurons in the IZ and dCP with disturbance in the Golgi orientation in the lower IZ, cell-cell adhesions between migrating neurons and radial fibers in the upper IZ, and cell motility during multipolar migration in the IZ and locomotion in the CP. The stalling of shArf4-knock-down neurons in the IZ was rescued by coexpressing shRNA-resistant Arf4, but not Arf5, despite the high similarity (∼90%) between the two proteins at the amino acid level (Volpicelli-Daley et al., 2005). These findings suggest that Arf4 has specific and nonredundant roles in radial migration. Since Arf4 was expressed in both radial glia and migrating neurons, we were unable to definitively conclude which cell type(s) (migrating neurons, radial glial cells, or both) is primarily responsible for the migration defects caused by Arf4 knock-down in this study. Our attempts to express dominant active or negative Arf4 mutants specifically in postmitotic migrating neurons under the control of the NeuroD promoter were unsuccessful because of the induction of apoptosis. However, we found that expression of shArf4 did not significantly affect cell cycle progression or the delamination of neural progenitor cells in the VZ. Furthermore, we failed to observe apparent morphologic abnormalities in radial glial fibers extending from the VZ to the pia (data not shown). Therefore, we believe that the migration defects caused by Arf4 knock-down primarily result from Arf4 dysfunction in migrating neurons, which should be confirmed in future studies by conditionally deleting the Arf4 gene in migrating cortical neurons using Arf4-floxed mice.

To gain insights into the role of Class II Arfs in migrating neurons, we first conducted immunohistological analyses to examine the subcellular localization of Class II Arfs. We found that both Arf4 and Arf5 were present in various organelles, including the Golgi apparatus (GM130), *trans*-Golgi network (TGN38A), retrograde transport vesicles to the TGN (VAMP4), and recycling endosomes (STX12), indicating the involvement of Class II Arfs in multiple membrane trafficking pathways. Furthermore, we observed that knock-down of Arf4 and Arf5 had overlapping but distinct effects on organelle morphology and distribution in migrating neurons. Knock-down of either Arf4 or Arf5 affected the sizes of GM130-immunoreactive, STX16-immunoreactive, and TGN38A-immunoreactive structures. Since Class II Arfs regulate vesicular transport from the Golgi to ER and within the Golgi through the recruitment of COPI (Hamlin et al., 2014), AP1 (Lowery et al., 2013), and GGAs (Lowery et al., 2013), the enlargement of the Golgi likely resulted from an imbalance between the influx and efflux caused by Class II Arf knock-down. Additionally, in HeLa cells, simultaneous knock-down of Arf1 and Arf4 was shown to inhibit retrograde transport of TGN38/46 from endosomes to the TGN (Nakai et al., 2013). On the other hand, knock-down effects on the size of VAMP2-immunoreactive, STX12-immunoreactive, and Rab11-immunoreactive puncta were specific for Arf4. VAMP4 and STX16 are SNARE partners that localize on transporting vesicles and their target TGN membrane and regulate retrograde transport to the TGN (Laufman et al., 2011), whereas STX12 is a component of the SNARE complex that localizes primarily on recycling endosomes (Prekeris et al., 1998) and Rab11 is a critical small GTPase for the recycling pathway to the plasma membrane (Ullrich et al., 1996). Therefore, it is tempting to speculate that Arf4 plays a distinct role from Arf5 in radial migration by controlling the balance of membrane trafficking in and out of the TGN via retrograde transport vesicles to the TGN and recycling endosomes from the TGN to the plasma membrane. However, it is also possible that Arf4 regulates neuronal migration by regulating the secretory pathway in the Golgi apparatus in an Arf5-independent manner. Further studies are needed to clarify these mechanisms.

Concerning cargo proteins that Arf4 regulates in migrating neurons, we demonstrated that knock-down of Arf4 resulted in the accumulation of N-cadherin in STX16-positive, VAMP4-positive, and TGN38-positive structures in migrating neurons, suggesting that Arf4 controls trafficking of *de novo* synthesized or endocytosed N-cadherin around the TGN. N-cadherin is a critical cell adhesion molecule that regulates various processes of radial migration, including those involved in cell proliferation and neurogenesis of radial glial progenitor cells in the VZ (Gil-Sanz et al., 2014), glial-independent somal translocation of early-born neurons (Franco et al., 2011), multipolar migration and multipolar-to-bipolar transition in the IZ (Jossin and Cooper, 2011), locomotion along radial glial fibers (Kawauchi et al., 2010), and glia-independent terminal translocation of late-born neurons in the uCP. We demonstrated that knock-down of Arf4 disturbed the Golgi orientation, cell-cell contact of bipolar neurons in the upper IZ, and cell motility during multipolar migration and locomotion, which were largely consistent with the phenotypes caused by N-cadherin dysfunctions. Furthermore, coexpression of N-cadherin with shArf4 partially rescued the migration defect caused by Arf4 knock-down. Taken together, these results suggest that Arf4 plays an important role in regulating radial migration by mediating the trafficking of N-cadherin from the TGN to the plasma membrane.

We have previously reported that Arf6 regulates multipolar migration through N-cadherin (Hara et al., 2016). However, knock-down effects of Arf4 and Arf6 differ in the morphology of organelles and subcellular localization of N-cadherin in migrating neurons. Arf6 knock-down led to the cytoplasmic accumulation of STX12-positive recycling endosomes in migrating neurons and disrupted the recycling of N-cadherin to the cell surface in cultured cortical neurons (Hara et al., 2016). On the other hand, Arf4 knock-down altered the morphology and distribution of various organelles including the Golgi, TGN, retrograde transport vesicles, and recycling endosomes, and induced accumulation of N-cadherin on the TGN and surrounding vesicles. Therefore, it is suggested that Arf4 and Arf6 regulate distinct steps of N-cadherin trafficking to the plasma membrane in migrating neurons.

It should be noted that the migration defect caused by Arf4 knock-down is not completely rescued by the coexpression of N-cadherin. In addition, knock-down of Arf4 did not affect multipolar-to-bipolar morphologic transition or leading process length, which was inconsistent with the previous findings observed by disruption of N-cadherin functions (Kawauchi et al., 2010; Martinez-Garay et al., 2016). These findings suggest that that Arf4 may regulate radial migration by trafficking other cargo proteins with N-cadherin. For instance, the β-amyloid precursor protein (β-APP) could be an attractive candidate for cargo regulated by the Arf pathway in migrating neurons. β-APP is a Type I transmembrane glycoprotein associated with the pathogenesis of familial Alzheimer’s disease and can function as an adhesion molecule that interacts with the APP family proteins and extracellular matrix proteins, such as heparan sulfate proteoglycans, laminin, collagen, and F-spondin (Narindrasorasak et al., 1991, 1992, 1995; Hoe and Rebeck, 2008). Notably, APP knock-down was previously shown to inhibit neuronal migration into the CP (Young-Pearse et al., 2007), similar to the phenotype induced by Arf4 knock-down. Furthermore, trafficking of APP to the cell surface and its localization to the Golgi/TGN are regulated in an Arf-dependent manner through the interaction of APP with Munc18-interacting proteins (MINTs) and phosphotyrosine binding (PTB) domain-containing coat proteins (Hill et al., 2003). Because MINTs can interact directly with GTP-bound Arf4 and function as a downstream effector of Arf4 (Hill et al., 2003), it is tempting to speculate that Arf4 may regulate radial migration by trafficking APP to the cell surface through interaction with MINTs. Another possible mechanism is Arf4-mediated ciliary transport. Arf4 has been proposed to mediate the sorting and transport of ciliary proteins from the TGN to the primary cilium (Deretic et al., 2005). Additionally, Arf4 plays a role in the trafficking of Notch components, such as Notch2 and presenilin-2, to basal bodies and/or primary cilia to promote epidermal differentiation (Deretic et al., 2005). The role of primary cilia in radial migration is still debated, but previous studies have identified 30 ciliopathy-related genes that impact cerebral cortex development, with knock-down of 17 genes resulting in disturbance of distinct steps of radial migration, including a transient multipolar stage in the lower IZ, multipolar-to-bipolar transition in the upper IZ, and radial glia-guided locomotion in the CP (Guo et al., 2015). It is therefore plausible to hypothesize that Arf4 regulates radial migration by facilitating the ciliary transport of these ciliopathy-related gene products. However, further investigation is required to confirm this hypothesis.

Lastly, mutations in the human genes for ARFGEF2 and ARF1 have been associated with cortical malformations, including periventricular nodular heterotopia and microcephaly, indicating that the ARFGEF2-Arf1 pathway is critical for cerebral cortical development (Sheen et al., 2004; Gana et al., 2022). Notably, a recent study reported a crosstalk cascade between Class II Arfs and Arf1 in the TGN: GBF1, a GEF for Class II Arfs, activates Arf4 and Arf5 at the TGN, where the resultant GTP-bound Class II Arfs interact with and recruit ARFGEF1/2 (Lowery et al., 2013), thereby initiating Arf1-dependent protein sorting and vesicle budding at the TGN. Therefore, it is attractive to speculate that Arf4 functions upstream of ARFGEF2-Arf1 signaling in radial migration. Further elucidation of the mechanisms by which Arf4 regulates radial migration may provide additional clues to understand the role of the ARFGEF2-Arf1 pathway in pathogenesis of human cortical malformation.

## Synthesis

Reviewing Editor: Lynda Erskine, University of Aberdeen Decisions are customarily a result of the Reviewing Editor and the peer reviewers coming together and discussing their recommendations until a consensus is reached. When revisions are invited, a fact-based synthesis statement explaining their decision and outlining what is needed to prepare a revision will be listed below. The following reviewer(s) agreed to reveal their identity: Takeshi Kawauchi.

Thank you for submitting your revised manuscript. The reviewers agreed that you had adequately addressed all of the concerns raised in the previous reviews and have no further comments. It was noted, however, that there are some typos in the manuscript (“marge” in Figure 2 for example). The second sentence of the significance sentence also seems to state the opposite of what intended (“However its molecular detail remained fully understood).

## Author Response

Major issues:

1. A single shRNA has been used for Arf4 and for Arf5. Although partial rescue of the migration defects is shown with an shRNA-resistant Arf4, it would be good to show a second shRNA showing similar defects and/or rescue experiments for morphology and migration speed to rule out off target effects.

(Response)

According to the reviewer’s comment, we prepared an independent shRNA against Arf4 (shArf4#2) (Fig. 3A) and examined its effect on neuronal migration by in utero electroporation. As shown in Fig. 4A and 4B, the second shRNA against Arf4 (shArf4#2) had basically the same effects on radial migration at E17 and P0, except for the absence of significant accumulation of transfected cells in the IZ at P0 compared with the phenotype with shArf4#1. Since the knockdown effect of shArf4#2 was milder than that of shArf4#1 (Fig. 3A), we believe that this discrepancy can be explained by the difference in the knockdown efficiency between the two shRNAs. Together with the result of the rescue experiment, we believe that the migration defect is caused by Arf4 knockdown but not by the off-target effect.

2. Regarding Arf5, in Figure 4 it can be seen that shRNA against Arf5 also has an effect on migration, with increased cell % in VZ and reduced in PCZ. However, in lines 302 - 304, the authors claim that “In contrast, knockdown of Arf5 did not disturb cortical layer formation at E17 and P0; further, the distribution of shArf5-transfected cells in each cortical layer was comparable with that of control cells”. How do the authors reconcile this claim with the observed differences? Could Arf5 play a role in terminal translocation?

(Response)

The description of preliminary results was mistakenly left in the text. As pointed out by the reviewer, Arf5 knockdown indeed had a subtle, but significant, effect on the distribution of transfected neurons in the upper cortical layer, suggesting that Arf5 may play a role in terminal translocation. We have corrected the previous description and suggested a possibility that Arf5 may play a role in terminal translocation in the revised Result section. On the other hand, the statistical significance of the effect of shArf5 on the distribution in the VZ, which was observed in the previous Fig. 4, has been eliminated when the data obtained with shArf4#2 were included in the statistical analysis.

3. In Figure 4, the Arf4-knockdown cells could not enter the PCZ. It is unclear why the authors divide the PCZ and upper CP. Because PCZ is a place that the terminal translocation occurs, this result may suggest that Arf4 depletion shows the terminal translocation defect. Seemingly, however, the Arf4-knockdown neurons were not stalled just beneath the PCZ, and therefore these neurons could not enter the PCZ due to defect in the locomotion mode of the migration. If the authors would not conclude that Arf4 is required for the terminal translocation, the PCZ should be combined with the upper CP. In general, the upper CP includes the PCZ.

(Response)

According to the reviewer’s comment, we combined the PCZ and upper CP as the upper CP, and then performed statistical analysis as shown in Fig. 4B, 4C, and 9E. As described above, there still remains to be a statistical significance of the effect of shArf5 on the distribution of transfected neurons in the uCP at P0 compared with that of the control shRNA (Fig. 4B). Thus, we have described the possibility of the functional involvement of Arf5 in terminal translocation in the revised Result section.

4. The authors claim that “Knockdown of Arf4 or Arf5 does not affect neuronal differentiation and cell proliferation” and that “quantification of the percentage of BrdU-incorporation in transfected cells in the VZ and SVZ revealed that knockdown of either Arf4 or Arf5 did not alter cell cycle progression in neural progenitor cells”. However, these claims could be considered an overstatement, as no detailed analysis of proliferation has been carried out. The experimental design will just show how many cells are in S-phase at that moment of the BrdU injection, but it won’t detect any differences in the numbers/proportions of RGCs, IPs and neurons. Furthermore, it is important to consider how long it will take for the shRNA to reduce the levels of Arf4/5. The validation of the shRNAs was performed in vitro, with cells collected 3 days after transfection. In this in vivo experiment, BrdU was administered 1 day after iuEP, and brains were fixed 15 min after the final BrdU injection. Has there been enough time to reduce Arf4/5 levels in RGC progenitors? Or will the shRNAs work primarily in IPs or even neurons?

Also relating to this topic, in Figure 5A, the images for shArf4 and shArf5 seem to show a different cellular distribution compared with the control, with more cells in the SVZ and IZ. Given the quantification, maybe a different image could be shown for the control?

In Fig. 5B, given the number and distribution of the data points, it seems difficult to make any claims regarding differences (or lack thereof) between conditions. What is the power of this comparisons?

(Response)

In this revision, we first examined whether the shRNA had started to reduce endogenous expression of the respective targets in the VZ, and demonstrated that the immunofluorescene intensity for the target Arf was significantly reduced compared with that in control sRNA-transfected cells in the VP even at 1day after transfection as shown in revised Figs. 5A and B (Arf4: Control, 1.0 {plus minus} 0.26, n=33 cells; shArf4#1, 0.53 {plus minus} 0.17, n=37 cells, P < 0.0001; Arf5: Control, 1.0 {plus minus} 0.51, n=21 cells; shArf5, 0.24 {plus minus} 0.16, n=21 cells, P < 0.0001). As pointed out by the reviewer, the present labeling experiments only represent cells that are in the S phase of the cell cycle at the moment of the BrdU injection. In this revision, in addition to BrdU labeling, we also examined the effect of Arf4/5 knockdown on the proportion of cells in the M phase in transfected neurons in the VZ by immunostaining with anti-phospho-histone H3. As shown in Fig. 5E, we demonstrated that there were no significant differences in the proportion of mitotic cells among control shRNA-, shArf4#1-, and shArf5-transfected cells in the VZ. Together, these results have strengthened the possibility that knockdown of Arf4 and Arf5 did not apparently affect the cell cycle progression of the ventricular progenitor cells.

According to the reviewer’s comment, we have replaced the previous Fig5A with more representative images showing no apparent differences in the distribution of transfected cells in the VZ, SVZ and IZ among the control, shArf4, and shArf5.

Concerning the data in Fig. 5B, we increased the sample number (n=5) and further confirmed that there were indeed significant differences in the proportion of BrdU-positive cells among control shRNA-, shArr4#1-, and shArf5-transfected cells in the VZ.

5. The conclusion in Figure 5C-5E is problematic. It is an understandable result that the multipolar morphology of the Arf4-knockdown cells is affected because the number of the multipolar processes was increased in Figure 5E. However, defects in the immature neurite pruning may also lead to increase of the multipolar process number. Considering that the immature neurite pruning requires proper endocytosis and membrane trafficking, the ratio of cells possessing both a leading process and the remaining immature neurites should be measured. Moreover, it is unclear whether the multipolar-to-bipolar transition is disturbed. If the transition would be suppressed, the position of Golgi would be changed as the result in Figure 5C. To elucidate this, the ratio of cells with a leading process, multipolar cells and round cells in the intermediate zone should be quantified. Together with these additional experiments, which step(s) of the neuronal morphological changes is disturbed should be discussed.

(Response)

According to the reviewer’s comment, we examined the effect of Arf4/5 knockdown on the multipolar-to-bipolar morphological transition in the upper IZ by classifying the morphology of transfected cells as multipolar, round, bipolar shapes. We found that Arf4/5 knockdown did not affect the multipolar-to-bipolar morphological transition per se as shown in Fig. 5H and J. These findings suggest that disturbed Golgi orientation was not the secondary effect of the disturbed morphological transition. However, we also noticed that the cell morphology of Arf4 knockdown cells appeared to be abnormal in that they possessed more filopodia-like, fine, short processes extending from their cell bodies and bases of leading processes as shown in Fig. 5I and 5K. These findings suggest that Arf4 regulates cell shape probably through actin cytoskeleton reorganization in migrating neurons, although it does not mediate multipolar-to-bipolar morphological transition per se. In this revision, we have provided new graphs showing the effects of Arf4/5 knockdown on multipolar-to-bipolar morphological transition in revised Fig. 5H and 5J, and described these findings in the Results section.

6. The characterization of the new anti-STX16 antibody is limited to a Western Blot with brain lysate. Although bands of the expected size are detected, a proper validation should include either KO tissue or at least knockdown of STX16.

(Response)

According to the reviewer’s comment, we tried to confirm the specificity of anti-Stx16 using a knockdown approach. However, we failed to obtain shRNAs that efficiently reduced the expression of exogenously transfected FLAG-Stx16 as shown below. In this revision, we have alternatively shown the specificity of the antibody by immunoblotting of lysates of mouse brain and HEK293T cells transfected with FLAG-STX16 (Fig. 7A). Furthermore, we have immunohistologically shown that this antibody labeled puncta partially overlapped and/or associated with TGN38A in migrating neurons (Fig. 7F) as previously reported (Mallard et al., 2002), which were completely eliminated by preaborption of the antibody with Stx16 (Fig. 7D-E).

7. What is the rationale for measuring fluorescence intensity for STX16, EEA1 and STX12, rather than number of puncta or size/morphology? What does fluorescence intensity represent in this case? Also, the images provided seem to have saturated pixels (both in Figure 7 and in Figure 8 for quantification of N-Cadherin). Was the FI measured on them?

(Response)

Because we had preliminary findings that knockdown of Arf4 or Arf5 did not have apparent effects on endogenous expression of GM130, Stx16, STX12, or TGN38A in transfected primary cortical neurons by immunoblotting, we presumed that the fluorescence intensity was likely to reflect the subcellular distribution pattern of each marker protein. In this revision, according to reviewer’s comment, we have shown the effects of knockdown of Arf4 or Arf5 on the immunoreactive area for each marker protein normalized by the cell body size in this revision. Consistent with the previous findings, we found that knockdown of Arf4 increased the immunoreactive area for STX16 and decreased those for STX12 and TGN38A without affecting that for EEA1.

Concerning the FI measurement, we always measured the immunoreactive intensity using images whose pixels were not saturated. In this revision, we have replaced the images in Fig8 and Fig9A with more representative images whose pixels are not saturated and that we analyzed.

8. Because Arf4 prefers to colocalize with VAMP4, rather than TGN38A (Figure 2), fluorescence intensity of VAMP4 in the Arf4-knockdown cells should be measured in Figure 7. Second, the fluorescence intensity of STX12 was decreased in the Arf4-knockdown neurons, which suggests that the recycling endosomes become smaller. The authors should confirm this result by the immunostaining with Rab11, another marker for the recycling endosomes.

(Response)

According to the reviewer’s comment, we have included the effects of Arf4/5 knockdown on the immunofluorescence areas of VAMP4 and Rab11 normalized by cell body areas in Fig. 8C and E. We found that the immunoreactive area for Rab11 was similarly reduced to those for STX12 by Arf4 knockdown (Fig. 8D, E). On the other hand, VAMP4-immunreactive areas were reduced by Arf4 knockdown (Fig. 8C). These results have further strengthened our conclusion that Arf4 knockdown affects the morphology and distribution of the TGN and endosomal compartments in and out of the TGN.

9. Quantification of the data in Figure 8B is required. The colocalization between N-cadherin and STX16 does not seem to be high in the Arf4-knockdown neurons. In that case, the colocalization of N-cadherin and VAMP4 or TGN38A in the Arf4-knockdown cells should also be quantified.

(Response)

According to reviewer’s comment, we quantitatively examined the colocalization of N-cadherin with Stx16, TGN38A, and VAMP4 in shArf4-transfected neurons. As a result, we have shown that N-cadherin was accumulated in STX16-, TGN38A-, and VAMP4-immunoreactive structures shown in revised Fig. 9B, suggesting that N-cadherin was accumulated around the TGN.

10. Regarding Figure 8A, if the cell surface levels of N-cadherin would be reduced, the leading process length would be shortened because a previous study indicates that knockdown of N-cadherin suppresses the leading process elongation. The leading process length of the Arf4-knockdown cells should be measured.

(Response)

The role of N-cadherin in the leading process length is still in debate. Shikanai et al. (2011) reported that N-cadherin-knockdown neurons possess a short and irregular leading process, whereas Martinez-Garay et al. (2016) showed that the leading process of migrating neurons in the CP became longer when cadherin functions were perturbed by overexpression of dominant negative cadherin2/4. In this revision, we measured the length of leading processes of Arf4 knockdown neurons, and found Arf4 knockdown did not have apparent effects on the leading process length compared with the control as shown in Fig. 9D. In addition, Martinez-Garay et al. (2016) reported that overexpression of dominant negative cadherin2/4 induced fine lateral branches from the leading process in migrating neuron. Consistent with this, we have shown that Arf4-knockdown neurons had more filopodia-like, short processes from their cell bodies and leading processes compared with the control as shown in Fig. 5I and K, suggesting that Arf4 may regulate the actin cytoskeleton dynamics to restrain the formation of side branches or to enhance their retraction in migrating neurons. Whether the effect of Arf4 knockdown on short process formation from the cell body and leading process in migrating neurons is N-cadherin-dependent or not remains to be elucidated in the future study.

11. There is no specific section in the Material and Methods addressing the statistical analyses performed. Instead, that information is provided in the figure legends. However, the information is very succinct, and there is for example no reference as to whether datasets were tested for normality or equality of variance before the statistical tests were used. Also, in cases where the control condition has been normalized to 1 and therefore lacks any variability, there is no indication of how that has been considered when applying the test.

(Response)

In this revision, we have provided detailed information on the statistical analyses performed in this study in Table 3.

Additional points:

1. The VZ, SVZ and IZ are normally not referred to as “layers”, as that term tends to be reserved for layer 1-6 of the (mature) cortex.

In this revision, we collectively referred the VZ, SVZ, and IZ to as cortical zones instead of layers

2. Figure 1: there is not much difference between panels B and D, and the arrows in D do not point to anything markedly different from B.

(Response)

According to the reviewer’s comment, we have removed the previous Fig. 1B and D in this revision.

3. Figure 2: What percentage of Arf4/5 signal co-localizes with the markers? Is there a significant amount that does not co-localize? If so, what would that signal represent?

(Response)

Unfortunately, we do not know the precise percentage of Arf4/5 signal co-localizes with the marker, since we did colocalization analyses using the 2D image. It is generally considered that GDP-bound inactive Arfs are cytosolic and translocate to the membrane fraction when they are activated and converted to GTP-bound form by GEFs. Therefore, it is possible that the fractions of Arf4/5 that are not colocalized with the markers correspond to their cytosolic GDP-bound form or the fractions localized to other organelles, such as lysosomes or mitochondria, which were not examined in this study. It should also be noted that organelle markers does not always visualize the complete organelle morphology, since they localize only to certain organelle microdomains. Therefore, it is also possible that Arf4/5 is closely associated, but does not overlap, with organelle markers even when Arf4/5 and markers are present on the same organelles.

4. How were the colocalization coefficients in Table 2 generated?

(Response)

In this revision, we described the detailed information on how the colocalization coefficients were measured using the ZEN software (Zeiss) in the Materials and Methods.

5. With respect to the migration defects observed with shRNA against Arf4, have the authors checked whether it is a permanent migration defect or a migration delay? This could be easily determined by checking at later stages (e.g. around P10).

(Response)

According to reviewer’s comment, we examined the effect of Arf4 knockdown on the cortical structure in the brain on postnatal day 10, which had been transfected with shArf4#1 on embryonic day 14, and found that knockdown of Arf4 caused a permanent migration defect rather than a transient migration delay as shown in Fig. 4F.

6. Figure 3: it would be good to quantify the intensity in the cells as well to get an idea of how much reduction there is in vivo, as some signal is still visible.

(Response)

In this revision, we examined the in vivo efficiency of Arf4/5 knockdown in transfected cell by quantifying the intensity of immunofluorescence in brain sections, and found that shRNA for Arf4 and Arf5 significantly reduced the immunofluorescence intensity for the respective proteins by 66% and　62%, respectively, in transfected neurons in brain sections.

7. Lines 330 - 334: it would make it easier to compare if the results for shControl and shArf4 and 5 were listed in the same order.

(Response)

In this revision, we have changed the description to compare the effect of shControl, shArf4, and shArf5 on BrdU incorporation in the same order.

8. Line 350: units are missing in the provided data and the control values should be included.

(Response)

In this revision, we have added the units (%) and the control value of the percentage of cells with the Golgi facing the pia.

9. Figure 5E: it would be helpful to include further examples of cells with multiple processes and to show how the processes were quantified. The >10 processes category is difficult to judge from the figure. How was the number of processes quantified? This is not explained in the Materials and Methods. Were several imaging planes used?

(Response)

We evaluated the process number using stack images. As further examples of the cell shape of Arf4-knockdown cells in were shown below, we consistently observed that shArf4-transfected neurons had more processes from their cell bodies. In this revision, we have provided the information on how we quantified the process number in the Materials and Methods section and also shown the processes that we had counted, by arrows in Fig. 5E.

10. Figure 7: No units are provided for the Golgi size or the TGN38A+ transgolgi network. Is this because of the normalization? Also, why was normalization applied in all cases and how was it performed, given that the control shows multiple values and a SEM? Are data normalized to the average of the control?

Drawing the neuronal outlines in the green channel images would be helpful to recognize which staining corresponds to the electroporated neurons (particularly in panel E).

There is also an issue with labelling of panel E.

(Response)

Because we measured the immunoreactive areas by pixels using Fiji software, there were no units for values. The reason for the normalization is to clearly show the effect of knockdown compared with the control. We have confirmed that normalization does not affect the conclusion at all, compared with the raw pixel data without normalization.

We have provided the outlines of transfected cells visualized by mCherry in revised Fig. 8A-G, and also corrected the labelling of panel in revised Fig. 8D (previous Fig. 7E).

11. Lines 402 and 403: the conclusion is very vague; which are those “shared and specific roles of class II Arfs in organelle integrity and vesicular trafficking in migrating neurons”, and how are they inferred from the quantification performed?

(Response)

In this revision, we have changed the description as follows; these results suggest that Arf4 plays overlapping but distinct roles from Arf5 in the morphology and/or distribution of endosomal compartments related to the retrograde transport into the TGN and recycling to the plasma membrane.

12. Figure 8A: The relative intensity of N-Cadherin fluorescence is quantified (relative to control). Does the term “relative” refer to this normalization? Same question regarding normalization as in Fig 7.

(Response)

In Fig.7 and Fig. 8A, we showed the intensity of organelle markers or N-cadherin in shArf4#1- or shArf5-transfected cells normalized by that in the control cells as “relative intensity”. In this revision, we have provided this information in the Materials and Methods section and Figure legends.

13. Figure 8C: the leading process of the shArf4 EP neuron looks different from the control (wider and less smooth). Was this a common phenotype? If so, could the authors comment on it?

Also, according to the figure legend, the contact index was calculated on a single image. However, leading processes usually span several confocal planes. How was the image plane chosen? What impact could this have on the results?

(Response)

As pointed out by the reviewer, the leading process of shArf4-transfected neurons looks abnormal in terms of their contour and width. The irregular and less smooth contour of shArf4-transfected neurons is likely to be ascribed to the increased filopodia-like short processes from the cell body (Fig. 5K) and leading process. In addition, we consistently observed that the width of the leading process was wider in shArf4-knockdown neurons compared with the control, although we did not quantitatively evaluate this effect. In this revision, we have described this phenotype in the Result section. We examine the effect of Arf4 knockdown on the contact between transfected cells and radial fibers using single image and described this information in the revised Materials and Methods section.

14. In Figure 6B, the migration speed of the multipolar cells in the IZ was measured. It is known that the locomoting neurons are also located in the IZ, as well as the CP. The authors should clearly mention whether cells possessing a leading process in the IZ were not counted in Figure 6B.

(Response)

We measured the migration speed of cells migrating in the lower IZ at E17, where most migrating neurons were multipolar. In the revision, we have described this condition in the Materials and Methods.

15. The authors used Cux1 as a neuronal marker in Figure 4. It also needs to stain with Tuj1, a more general marker for differentiated neurons.

(Response)

According to reviewer’s comment, we have shown the expression of Tuji1 in shArf4-transfected neurons stalled in the IZ in Fig. 4D, further confirming that Arf4 knockdown did not affect neuronal differentiation.

17. Please check the manuscript carefully for typographical errors.

For example, Page 19, Fig. 7 should be changed into Fig. 8. Line 423, EFGP should be changed into EGFP.

Figure 7E, STX13 should be changed into STX12.

(Response)

We thank the reviewers for pointing out our careless typos. In this revision, we have corrected typographical errors as carefully as possible.

Mallard F, Tang BL, Galli T, Tenza D, Saint-Pol A, Yue X, Antony C, Hong W, Goud B, Johannes L (2002) Early/recycling endosomes-to-TGN transport involves two SNARE complexes and a Rab6 isoform. J Cell Biol 156:653-664.
